# Electronic cigarette use during pregnancy and the risk of adverse birth outcomes: A cross-sectional surveillance study of the US Pregnancy Risk Assessment Monitoring System (PRAMS) population

**DOI:** 10.1371/journal.pone.0287348

**Published:** 2023-10-24

**Authors:** Lin Ammar, Hilary A. Tindle, Angela M. Miller, Margaret A. Adgent, Hui Nian, Kelli K. Ryckman, Mulubrhan Mogos, Mariann R. Piano, Ethan Xie, Brittney M. Snyder, Abhismitha Ramesh, Chang Yu, Tina V. Hartert, Pingsheng Wu

**Affiliations:** 1 Vanderbilt University School of Medicine, Nashville, Tennessee, United States of America; 2 Department of Medicine, Vanderbilt University Medical Center, Nashville, Tennessee, United States of America; 3 The Vanderbilt Center for Tobacco, Addiction and Lifestyle, Vanderbilt University Medical Center, Nashville, Tennessee, United States of America; 4 Geriatric Research Education and Clinical Centers, Veterans Affairs Tennessee Valley Healthcare System, Nashville, Tennessee, United States of America; 5 Division of Population Health Assessment, Tennessee Department of Health, Nashville, Tennessee, United States of America; 6 Department of Health Policy, Vanderbilt University Medical Center, Nashville, Tennessee, United States of America; 7 Department of Biostatistics, Vanderbilt University Medical Center, Nashville, Tennessee, United States of America; 8 Department of Epidemiology and Biostatistics, Indiana University School of Public Health—Bloomington, Bloomington, IN, United States of America; 9 Department of Epidemiology, University of Iowa, Iowa City, IA, United States of America; 10 Vanderbilt University School of Nursing, Nashville, Tennessee, United States of America; 11 Division of Biostatistics, Department of Population Health, New York University Langone Health, New York University Grossman School of Medicine, New York, New York, United States of America; 12 Department of Pediatrics, Vanderbilt University Medical Center, Nashville, Tennessee, United States of America; Tulane University School of Public Health and Tropical Medicine, UNITED STATES

## Abstract

**Background:**

Research on health effects and potential harms of electronic cigarette (EC) use during pregnancy is limited. We sought to determine the risks of pregnancy EC use on pregnancy-related adverse birth outcomes and assess whether quitting ECs reduces the risks.

**Methods:**

Women with singleton live births who participated in the US Pregnancy Risk Assessment Monitoring System (PRAMS) survey study 2016–2020 were classified into four mutually exclusive groups, by their use of ECs and combustible cigarettes (CCs) during pregnancy: non-use, EC only use, CC only use, and dual use. We determined the risk of preterm birth, low birth weight, and small-for-gestational-age (SGA) by comparing cigarette users to non-users with a modified Poisson regression model adjusting for covariates. In a subset of women who all used ECs prior to pregnancy, we determined whether quitting EC use reduces the risk of preterm birth, low birth weight, and SGA by comparing to those who continued its use. All analyses were weighted to account for the PRAMS survey design and non-response rate.

**Results:**

Of the 190,707 women (weighted N = 10,202,413) included, 92.1% reported cigarette non-use, 0.5% EC only use, 6.7% CC only use, and 0.7% dual use during pregnancy. Compared with non-use, EC only use was associated with a significantly increased risk of preterm birth (adjusted risk ratio [aRR]: 1.29, 95% confidence interval [CI]: 1.00, 1.65) and low birth weight (aRR: 1.38, 95%CI: 1.09, 1.75), but not SGA (aRR: 1.04, 95%CI: 0.76, 1.44). Among 7,877 (weighted N = 422,533) women EC users, quitting use was associated with a significantly reduced risk of low birth weight (aRR: 0.76, 95%CI: 0.62, 0.94) and SGA (aRR: 0.77, 95%CI: 0.62, 0.94) compared to those who continued to use ECs during pregnancy.

**Conclusions:**

Pregnancy EC use, by itself or dual use with CC, is associated with preterm birth and low birth weight. Quitting use reduces that risk. ECs should not be considered as a safe alternative nor a viable gestational smoking cessation strategy.

## Introduction

Preterm birth and low birth weight are the most common adverse outcomes of pregnancy imposing significant economic and psychological burden on families and society [1–3]. Preterm birth and low birth weight are important causes of perinatal mortality and both short- and long-term infant and childhood morbidity [2, 4]. Decades of research has established maternal smoking during pregnancy as a major risk factor for preterm birth and low birth weight [5–7]. What remains unclear, however, is whether electronic cigarette (EC) use during pregnancy is associated with these adverse birth outcomes [8].

Since their introduction in the U.S. market in 2007, ECs have been marketed as a safer and healthier alternative to combustible cigarettes (CCs) [9]. Women smokers who are pregnant or planning to become pregnant often view ECs as less harmful to the fetus and an option for smoking cessation [10–14]. The U.S. Food and Drug Administration recently authorized the marketing of several electronic nicotine delivery system products, which may be misinterpreted as an endorsement about the safety of these products [15]. The 2022 Cochrane review concluded that nicotine-containing ECs are effective for smoking cessation [16]. However, research on health effects and potential harms of EC use during pregnancy is limited and has been identified as one of the research gaps by the 2021 U.S. Preventive Services Task Force (USPSTF) [8]. While the mechanisms of fetal harm from EC use during pregnancy are not known, they may be mediated via exposure to volatile organic compounds, heavy metals, carcinogens, nicotine, and carrier agents, among others [17]. EC vapor, independent of nicotine, hinders the function of trophoblasts in the placenta leading to complications in placental structuring and could lead to preeclampsia, early miscarriage, premature birth and possibly even maternal or fetal death [18]. Further, instead of switching to complete EC only use, women who use ECs intending to quit smoking may end up using both products (dual use) [9, 14].

The objectives of this study, in direct response to USPSTF call, were to investigate the risk of EC use during pregnancy on adverse birth outcomes of preterm birth, low birth weight, and small-for-gestational-age (SGA), and to assess whether quitting EC use in pregnancy reduces these health risks. Results can contribute to a critical growing body of knowledge on the health effects of EC use during pregnancy and the benefits of quitting, and will inform the development of clinical guidelines and public health interventions for tobacco control in pregnancy.

## Materials and methods

### Data source

We studied women who had singleton live births between 2016–2020 and participated in the Centers for Diseases Control and Prevention (CDC) Pregnancy Risk Assessment Monitoring System (PRAMS) survey, an ongoing public health surveillance survey of women who have had a recent live birth [19]. PRAMS contacts women 2–6 months post delivery for population-based and site-specific information on maternal attitudes and experiences before, during, and shortly after pregnancy. PRAMS additionally contains a wealth of demographic and medical information through linked birth certificates. Detailed methods regarding the PRAMS study design have been described elsewhere [20]. PRAMS has a minimum overall response rate threshold policy for the release of data for each year. Our analysis included data of a total 195 study-years from 48 sites that met the established response rate threshold criteria (S1 Table). The study is deemed as a secondary data analysis for which consent is not required. We have no access to information that could identify individual participants during or after data collection. The study protocol was approved by the CDC PRAMS Working Group and the Vanderbilt University Institutional Review Board (#191880).

### Tobacco product (EC and CC) use around pregnancy

We focus on tobacco products of EC and CC use around pregrancy. Information regarding women’s daily average amount of CC use in the 3 months before pregnancy, the last 3 months of pregnancy, and the 2–6 months post-delivery has been collected since the start of the PRAMS survey in 1988 (S2 Table). PRAMS introduced questions on ECs in the 2016 survey. EC and other electronic nicotine products (such as vape pens, e-hookahs, hookah pens, e-cigars, e-pipes) are defined as battery-powered devices that use nicotine liquid rather than tobacco leaves and produce vapor instead of smoke [19]. Women’s frequency of EC use in the 3 months before pregnancy and the last 3 months of pregnancy has been collected (S2 Table).

### Preterm birth, low birth weight, and SGA

The categorical variables of gestational age in weeks (≤27, 28–33, 34–36, 37–42, 43+ weeks), birth weight grouped per 250-gram intervals (0 to 7,000 grams), and SGA at the tenth percentile (birth weight lower than the tenth percentile of the population defined by gestational age in weeks, race/ethnicity, and infant sex) were available in the PRAMS data from the linked birth certificate (27). Our primary outcomes of interest thus included preterm birth (gestational age < 37 weeks), low birth weight (< 2,500 grams), and SGA.

### Covariates

Demographic, behavior, and health-related characteristics captured from the survey or linked birth certificate that are associated with maternal tobacco product use and/or birth outcomes were studied. Covariates included maternal age at delivery (≤17, 18–24, 25–29, 30–34, ≥35 years), maternal race/ethnicity (non-Hispanic White, non-Hispanic Black, Hispanics, and Other/Unknown), maternal education (some high school education or less, high school graduate, some college education, college graduate or more), marital status (married, other), household income (≤$20,000, $20,001-$40,000, $40,001-$85,000, ≥$85,001), prenatal participating in the Speical Supplemental Nutrition Program for Women, Infants, and Children (WIC) (no, yes), pregnancy intention (intended, unintended, unsure), the Kotelchuck index of prental care adequacy (inadequate, intermediate, adequate, adequate plus), commencement of prenatal care in the first trimester (no, yes), parity (primiparous, 2, ≥3), history of preterm birth (no, yes), maternal pre-pregnancy body mass index (BMI) (underweight: <18.5, normal weight: 18.5–24.9, over weight: 25.0–29.9, obese ≥30.0 kg/m^2^), pre-pregnancy multivitamin use frequency per week (never, 1–3 times, 4–6 times, everyday), pre-pregnancy alcoholic drink consumption per week (no, <1 drink, 1–7 drinks, ≥8 drinks), delivery method (vaginal, assisted, c-section), residency, and year of delivery (2016–2020).

### Study design and analysis

**Fig 1:**
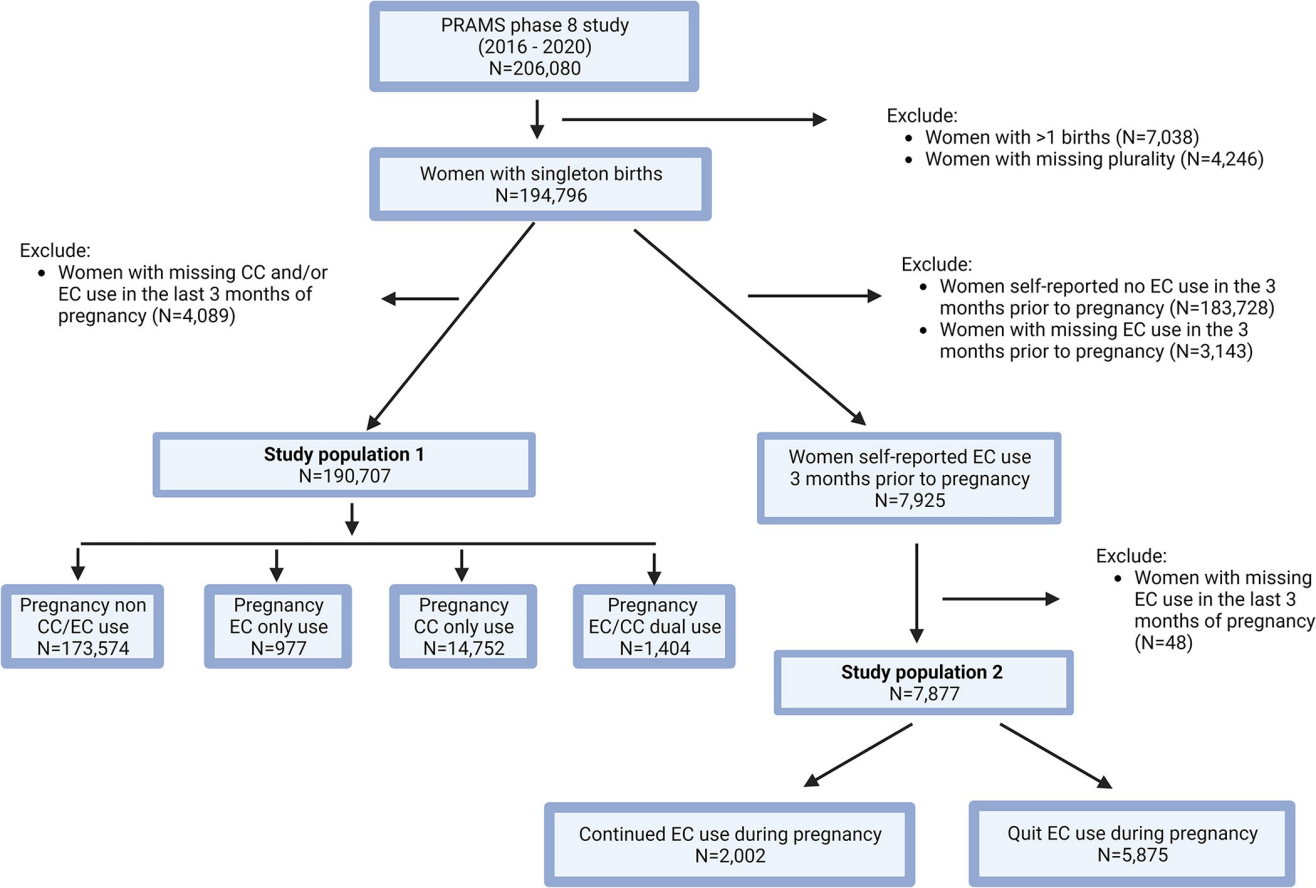
Flow diagram of the study populations. Numbers presented are unweighted sample size.

We assessed the risk of pregnancy EC use in two analyses. The first analysis aimed to address the question of whether pregnancy EC use increases the risk of adverse birth outcomes. The analysis included 190,707 (weighted N = 10,202,413) women with live singleton births who reported their EC and CC use in the last three months of pregnancy (study population one). Women were classified into mutually exclusive groups: non-use, EC only use, CC only use, and dual use (Fig 1). A modified Possion regression was performed to assess the relative risk of preterm birth, low birth weight, and SGA by comparing EC only use, CC only use, and dual use to non-use adjusting for covariates. As a sensitivity analysis, a proportional odds model was performed with categorical gestational age (≤27, 28–33, 34–36, 37–42, 43+ weeks) and categorical birth weight (extreme low birth weight <1,000 grams, very low birth weight: 1,000 - <1,500 grams, low birth weight: 1,500 - <2,500 grams, normal birth weight: > = 2,500 grams) as outcomes of interest. To better understand the association between EC use and low birth weight, we assessed the association in 1) a sensitivity analysis additionally adjusting for gestational age, and 2) a subgroup analysis of women who had a term birth (≥37 weeks).

The second analysis aimed to answer the question of whether quitting EC use during pregnancy reduces the risk of adverse birth outcomes. The analysis is limited to women who self-reported use of ECs three months prior to pregnancy, regardless of their CC use status (Fig 1, study population two). We excluded women with missing EC use in the last three months of pregnancy. Women EC users (unweighted N = 7,877, weighted N = 422,533) were then classified into those who self-reported continuing EC use and those who reported quitting use during pregnancy (Fig 1). The risk of preterm birth, low birth weight, and SGA between women of continuing use and quitting use was compared using a modified Poisson regression, adjusting for the number of CCs used before and during pregnancy (no use, <10, 11–20, >20) as well as the above covariates. The association was further examined in a subset of women who did not use CCs both prior to and during pregnancy.

We implemented a complete case analysis approach in above regression modeling. Subjects missing one or more covariates were dropped from the analysis. As a sensitivity analysis, we performed multiple imputation and repeated all analyses conducted. For each analysis, ten imputed data sets were generated using aregImpute function in the Hmisc package [21], and the weighted analysis results from each of the ten imputed data sets were combined using MIcombine function in the mitools package [22].

All analyses were weighted to account for the PRAMS survey design and non-response rate and were performed using STATA software, version 16.1 (StataCorp LLC, College station, Texas) and R software version 4.2.1 [23]. A p-value less than 0.05 was considered statistically significant.

## Results

Of 206,080 women (weighted population 10,630,861) who participated in the 2016–2020 PRAMS survey, we excluded 7,038 women who had multiple births and 4,246 women with missing information on plurality. We further excluded 4,089 women with missing information on EC and/or CC use in the last three months of pregnancy to leave study population one as 190,707 women (weighted N = 10,202,413). Study population two included women who self-reported EC use prior to pregnancy, regardless of their status of CC use (unweighted N = 7,877, weighted N = 422,533) (Fig 1).

Table 1 presents the unweighted sample size and the weighted prevalence (95% confidence interval [CI]) of women with a recent live singeton birth stratified by the status of self-reported cigarette use in the last three months of pregnancy. Overall, 92.1% (95%CI: 91.9%, 92.3%) women reported cigarette non-use, 0.5% (95%CI: 0.5%, 0.6%) reported EC only use, 6.7% (95%CI: 6.5%, 6.9%) reported CC only use, and 0.7% (95%CI: 0.6%, 0.8%) reported dual use during the last three months of pregnancy. Fifty-seven percent of women in the weighted sample were non-Hispanic White, 15% were non-Hispanic Black, 19% were Hispanics, and 9% were other race/ethnic categories. Compared with women who were non-users, women who used tobacco products, either ECs and/or CCs, were more likely to be non-Hispanic White, younger in age, less educated and have a lower household income. Women who used ECs and/or CCs were also less likely to be married, have an intended pregnancy, have at least “adequate” prenatal care, initiate prenatal care in the first trimester of pregnancy, and take multivitamins prior to pregnancy. They were more likely to receive federal food and nutritional assistance. Among women who reported use of tobacco products, EC only users were more likely to be younger, more educated, be married, have a higher total family income, have no previous live births, have at least “adequate” prenatal care and initiate care during the first trimester compared to CC only users; dual users were predominantly non-Hispanic White, had a lower total family income, had no previous live births, and had a lower pre-pregnancy BMI compared with CC only users (Table 1).

**Table 1:** Maternal demographic, behavioral, and health condition related characteristics of women who gave live singleton births in 2016–2020 and participated in the PRAMS survey.

Characteristics	Non-use (N = 173,574)^a^		EC only use (N = 977)^a^		CC only use (N = 14,752) ^a^		Dual use (N = 1,404)^a^		Total (N = 190,707)^a^	
	No.^a^	% (95%CI)^b^	No.^a^	% (95%CI)^b^	No.^a^	% (95%CI)^b^	No.^a^	% (95%CI)^b^	No.^a^	% (95%CI)^b^
Maternal age at delivery (year)										
≤24	39,396	22.2 (21.9, 22.5)	388	40.7 (35.8, 45.7)	4,194	29.9 (28.7, 31.2)	464	34.2 (30.2, 38.5)	44,442	22.9 (22.6, 23.2)
25–29	49,640	28.6 (28.3, 29.0)	283	24.8 (20.9, 29.1)	4,799	34.1 (32.8, 35.4)	439	32.2 (28.2, 36.4)	55,161	29.0 (28.7, 29.3)
30–34	51,594	30.1 (29.8, 30.5)	194	23.2 (19.2, 27.9)	3,649	23.2 (22.0, 24.3)	331	23.5 (20.1, 27.2)	55,768	29.6 (29.3, 29.9)
≥35	32,938	19.0 (18.7, 19.3)	111	11.3 (8.6, 14.7)	2,109	12.8 (11.9, 13.7)	170	10.2 (8.0, 12.9)	35,328	18.5 (18.2, 18.8)
Maternal race/ethnicity										
Non Hispanic White	76,485	55.3 (55.0, 55.6)	606	78.1 (74.1, 81.6)	7,918	72.6 (71.4, 73.8)	966	85.8 (83.1, 88.2)	85,975	56.8 (56.5, 57.1)
Non Hispanic Black	31,550	15.2 (15.0, 15.5)	86	6.0 (4.2, 8.5)	2,733	14.2 (13.3, 15.2)	117	4.0 (2.8, 5.7)	34,486	15.1 (14.8, 15.3)
Hispanic	36,033	20.0 (19.7, 20.3)	130	10.1 (7.6, 13.4)	1,075	6.4 (5.7, 7.1)	95	4.7 (3.4, 6.5)	37,333	18.9 (18.6, 19.2)
Other	28,309	9.5 (9.3, 9.7)	152	5.8 (4.4, 7.7)	2,925	6.7 (6.2, 7.3)	217	5.5 (4.2, 7.2)	31,603	9.3 (9.1, 9.4)
Maternal education										
Some high school education or less	19,549	10.9 (10.7, 11.2)	142	15.1 (11.6, 19.4)	3,595	23.8 (22.6, 25.0)	337	23.7 (20.1, 27.7)	23,623	11.9 (11.7, 12.2)
High school graduate	39,477	23.5 (23.2, 23.8)	372	37.4 (32.7, 42.3)	6,023	41.8 (40.4, 43.1)	562	41.5 (37.3, 45.8)	46,434	24.9 (24.6, 25.3)
Some college education	48,968	26.5 (26.2, 26.8)	356	36.5 (31.8, 41.4)	4,379	30.2 (29.0, 31.5)	432	30.5 (26.8, 34.6)	54,135	26.8 (26.5, 27.1)
College graduate or more	64,052	39.1 (38.7, 39.4)	99	11.1 (8.2, 14.7)	599	4.2 (3.7, 4.8)	62	4.3 (2.9, 6.2)	64,812	36.3 (36.0, 36.7)
Marital status										
Married	107,641	64.2 (63.9, 64.5)	344	38.1 (33.3, 43.1)	3,885	28.1 (26.9, 29.3)	381	26.2 (22.6, 30.0)	112,251	61.4 (61.0, 61.7)
Other	65,842	35.8 (35.5, 36.1)	628	61.9 (56.9, 66.7)	10,823	71.9 (70.7, 73.1)	1,016	73.8 (70.0, 77.4)	78,309	38.6 (38.3, 39.0)
Household income ($)										
≤20,000	42,281	24.2 (23.8, 24.5)	397	41.2 (36.1, 46.4)	8,369	58.3 (56.9, 59.7)	836	63.4 (59.1, 67.5)	51,883	26.8 (26.5, 27.2)
20,001–40,000	33,924	20.5 (20.2, 20.8)	251	24.0 (20.0, 28.5)	3,190	25.0 (23.8, 26.3)	312	21.7 (18.4, 25.4)	37,677	20.8 (20.5, 21.2)
40,001–85,000	39,210	25.0 (24.6, 25.3)	176	24.1 (19.6, 29.1)	1,611	13.2 (12.2, 14.1)	144	11.2 (8.8, 14.2)	41,141	24.1 (23.8, 24.4)
≥85,001	43,231	30.3 (30.0, 30.7)	79	10.8 (8.1, 14.3)	408	3.6 (3.1, 4.1)	36	3.7 (2.3, 5.8)	43,754	28.2 (27.9, 28.6)
Maternal WIC program participation										
No	62,128	66.6 (66.3, 67.0)	469	51.0 (45.9, 56.0)	8,849	39.8 (38.5, 41.2)	832	38.9 (34.7, 43.2)	72,278	64.6 (64.2, 64.9)
Yes	109,079	33.4 (33.0, 33.7)	492	49.0 (44.0, 54.1)	5,668	60.2 (58.8, 61.5)	548	61.1 (56.8, 65.3)	115,787	35.4 (35.1, 35.8)
Pregnancy intention										
Intended	102,184	60.1 (59.8, 60.5)	399	40.7 (35.9, 45.7)	5,077	34.7 (33.4, 36.0)	468	33.6 (29.7, 37.8)	108,128	58.1 (57.8, 58.5)
Unintended	42,374	24.2 (23.9, 24.6)	337	34.1 (29.6, 39.0)	4,986	34.9 (33.6, 36.2)	498	36.6 (32.6, 40.9)	48,195	25.1 (24.8, 25.4)
Unsure	29,016	15.6 (15.4, 15.9)	241	25.2 (20.9, 30.0)	4,689	30.4 (29.2, 31.7)	438	29.8 (26.0, 33.8)	34,384	16.8 (16.5, 17.0)
Kotelchuck index										
Inadequate	20,042	11.9 (11.7, 12.2)	138	11.2 (8.7, 14.2)	3,360	21.5 (20.4, 22.7)	351	24.3 (20.6, 28.4)	23,891	12.6 (12.4, 12.9)
Intermediate	17,467	10.8 (10.5, 11.0)	103	12.0 (8.7, 16.4)	1,706	11.0 (10.2, 11.9)	170	12.1 (9.6, 15.3)	19,446	10.8 (10.6, 11.0)
Adequate	74,101	46.1 (45.7, 46.4)	370	41.1 (36.2, 46.2)	4,758	37.4 (36.0, 38.7)	425	36.1 (32.0, 40.3)	79,654	45.4 (45.0, 45.7)
Adequate plus	56,972	31.2 (30.9, 31.6)	332	35.7 (31.0, 40.7)	4,499	30.1 (28.8, 31.3)	424	27.5 (23.9, 31.3)	62,227	31.2 (30.8, 31.5)
Prenatal care started in the 1^st^ trimester of pregnancy										
No	148,376	11.5 (11.3, 11.8)	790	14.4 (11.4, 18.1)	10,916	19.6 (18.6, 20.7)	1,001	23.0 (19.6, 26.9)	161,083	12.1 (11.9, 12.4)
Yes	20,165	87.7 (87.5, 88.0)	148	84.1 (80.2, 87.3)	3,130	78.5 (77.4, 79.6)	332	75.0 (71.0, 78.6)	23,775	87.0 (86.8, 87.3)
No prenatal care	1,331	0.7 (0.7, 0.8)	15	1.5 (0.6, 3.3)	341	1.9 (1.5, 2.2)	33	1.9 (1.0, 3.8)	1,720	0.8 (0.8, 0.9)
Parity										
Primiparous	70,428	40.0 (39.6, 40.3)	424	44.6 (39.7, 49.7)	4,019	27.1 (25.9, 28.3)	464	36.0 (31.9, 40.4)	75,335	39.1 (38.7, 39.4)
2	54,731	33.2 (32.8, 33.5)	289	30.1 (25.8, 34.8)	4,144	31.0 (29.7, 32.3)	389	26.7 (23.2, 30.4)	59,553	33.0 (32.6, 33.3)
≥3	48,073	26.9 (26.5, 27.2)	260	25.3 (21.2, 29.8)	6,557	41.9 (40.6, 43.3)	546	37.3 (33.3, 41.5)	55,436	27.9 (27.6, 28.3)
History of preterm birth										
No	7,555	96.7 (96.6, 96.9)	58	95.5 (93.3, 96.9)	1,316	93.3 (92.6, 93.9)	104	95.5 (93.8, 96.7)	9,033	96.5 (96.4, 96.6)
Yes	165,666	3.3 (3.1, 3.4)	918	4.5 (3.1, 6.7)	13,393	6.7 (6.1, 7.4)	1,297	4.5 (3.3, 6.2)	181,274	3.5 (3.4, 3.6)
Pre-pregnancy BMI										
Underweight (<18.5 kg/m^2^)	5,136	2.9 (2.8, 3.1)	59	4.9 (3.3, 7.2)	740	4.8 (4.3, 5.4)	89	6.6 (4.7, 9.3)	6,024	3.1 (3.0, 3.2)
Normal (18.5–24.9 kg/m^2^)	71,108	43.1 (42.8, 43.5)	377	38.7 (33.8, 43.8)	5,299	36.6 (35.3, 37.9)	578	41.6 (37.3, 45.9)	77,362	42.7 (42.3, 43.0)
Overweight (25.0–29.9 kg/m^2^)	44,822	26.5 (26.2, 26.8)	237	26.9 (22.7, 31.6)	3,447	24.7 (23.5, 25.9)	318	26.5 (22.8, 30.7)	48,824	26.4 (26.1, 26.7)
Obese (30.0+ kg/m^2^)	48,767	27.4 (27.1, 27.8)	282	29.5 (25.1, 34.3)	4,808	33.9 (32.6, 35.3)	372	25.3 (21.8, 29.1)	54,229	27.8 (27.5, 28.2)
Pre-pregnancy multivitamin use per week										
Never	86,502	49.4 (49.0, 49.8)	661	72.0 (67.4, 76.1)	10,823	74.2 (73.0, 75.4)	1,028	75.3 (71.6, 78.8)	99,014	51.4 (51.0, 51.7)
1–3 times	12,884	7.3 (7.1, 7.5)	81	7.0 (5.0, 9.6)	883	5.6 (5.0, 6.2)	93	6.5 (4.7, 8.9)	13,941	7.2 (7.0, 7.4)
4–6 times	10,921	6.3 (6.1, 6.5)	32	2.3 (1.4, 3.7)	442	3.0 (2.5, 3.5)	54	2.8 (1.8, 4.4)	11,449	6.0 (5.9, 6.2)
Everyday	62,338	37.0 (36.6, 37.4)	196	18.8 (15.2, 23.0)	2,548	17.3 (16.3, 18.3)	226	15.3 (12.6, 18.6)	65,308	35.4 (35.1, 35.8)
Pre-pregnancy alcoholic drinks per week										
No	78,460	43.1 (42.8, 43.5)	374	40.9 (36.0, 46.0)	6,415	41.8 (40.5, 43.2)	537	39.4 (35.3, 43.7)	85,786	43.0 (42.6, 43.3)
< 1 drink	45,245	27.4 (27.1, 27.7)	235	23.4 (19.4, 28.0)	3,640	26.5 (25.3, 27.8)	406	31.2 (27.4, 35.4)	49,526	27.3 (27.0, 27.7)
1 to 7 drinks	44,479	27.0 (26.7, 27.4)	283	29.3 (25.0, 34.0)	3,762	25.9 (24.7, 27.2)	341	23.6 (20.0, 27.5)	48,865	26.9 (26.6, 27.3)
> = 8 drinks	4,181	2.5 (2.4, 2.6)	76	6.3 (4.5, 8.8)	800	5.7 (5.1, 6.4)	105	5.8 (4.3, 7.8)	5,162	2.7 (2.6, 2.8)
Delivery method										
Vaginal	113,065	66.8 (66.5, 67.2)	634	67.8 (63.0, 72.2)	9,325	64.6 (63.2, 65.9)	896	65.6 (61.3, 69.6)	123,920	66.7 (66.3, 67.0)
Assisted	5,450	3.2 (3.1, 3.4)	29	2.8 (1.5, 5.2)	346	2.7 (2.3, 3.2)	33	2.8 (1.6, 4.9)	5,858	3.2 (3.1, 3.3)
C-section	54,923	29.9 (29.6, 30.3)	313	29.4 (25.1, 34.1)	5,067	32.8 (31.5, 34.1)	474	31.6 (27.7, 35.8)	60,777	30.1 (29.8, 30.5)
Year of delivery										
2016	29,770	19.0 (18.9, 19.1)	135	13.7 (10.6, 17.5)	2,691	19.7 (18.7, 20.9)	270	18.5 (15.3, 22.1)	32,866	19.0 (18.9, 19.1)
2017	32,187	18.2 (18.1, 18.3)	142	14.8 (11.7, 18.4)	3,036	20.5 (19.5, 21.5)	250	18.5 (15.5, 22.0)	35,615	18.3 (18.3, 18.4)
2018	37,625	20.7 (20.5, 20.8)	192	18.8 (15.3, 22.8)	3,309	22.3 (21.2, 23.4)	306	22.2 (18.9, 25.9)	41,432	20.8 (20.7, 20.8)
2019	38,506	22.1 (21.9, 22.2)	233	23.5 (19.3, 28.2)	3,022	20.2 (19.2, 21.4)	305	21.0 (17.6, 24.7)	42,066	21.9 (21.8, 22.0)
2020	35,486	20.1 (20.0, 20.2)	275	29.3 (25.0, 34.1)	2,694	17.2 (16.3, 18.3)	273	19.9 (16.7, 23.5)	38,728	20.0 (19.9, 20.0)

^a^aighted sample size.

^b^Weighted prevalence and corresponding confidence interval (expressed as a percentage).

**Fig 2:**
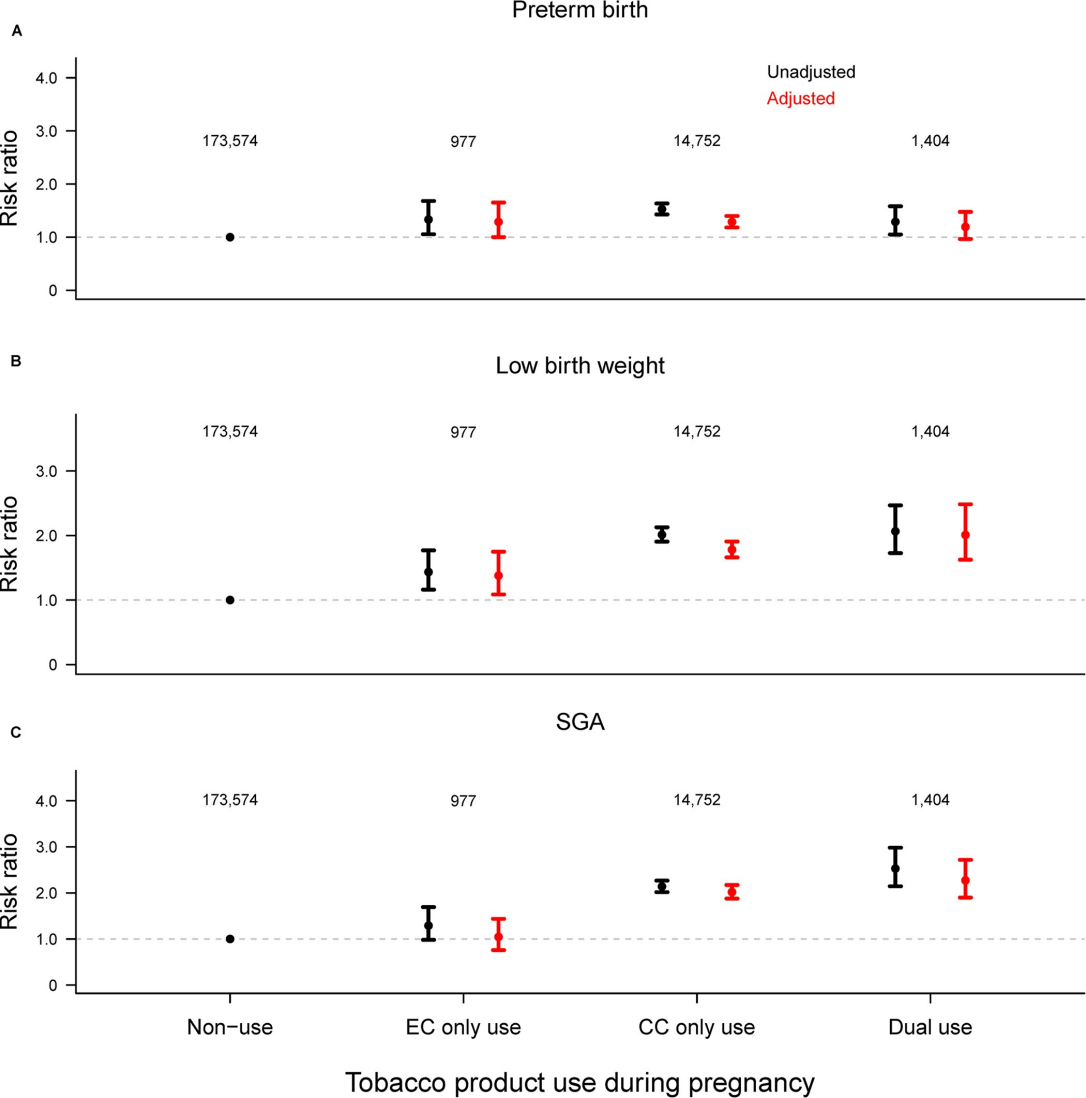
Risk of (A) preterm birth, (B) low birth weight, and (C) SGA comparing women of EC only use, CC only use, and dual use during pregnancy to women who were non-users. Numbers presented are unweighted sample sizes in each group. The multivariable modified Poisson regression (red) was adjusted for maternal age at delivery, maternal race/ethnicity, maternal education, marital status, household income, prenatal federal nutritional assistance, pregnancy intention, the Kotelchuck index, initiation of prenatal care in the first trimester, pre-pregnancy multivitamin use, pre-pregnancy alcoholic drinking frequency, parity, history of preterm birth, maternal pre-pregnancy BMI, maternal residency, and year of delivery.

The weighted frequency of preterm birth <37 weeks was 8.0% (95%CI: 7.8%, 8.2%), low birth weight <2,500 grams 6.5% (95%CI: 6.4%, 6.6%), and SGA 9.7% (95%CI: 9.4%, 9.9%) (Table 2). Compared with non-use, EC only use was associated with a significantly increased risk of preterm birth (adjusted risk ratio [aRR]: 1.29, 95%CI: 1.00, 1.65) and low birth weight (aRR: 1.38, 95%CI: 1.09, 1.75) in both the univariate and adjusted analyses, but not SGA (aRR: 1.04, 95%CI: 0.76, 1.44). Dual use, when compared with non-use, was associated with an increased risk of preterm birth (aRR: 1.19, 95%CI: 0.97, 1.48), low birth weight (aRR: 2.01, 95%CI: 1.63, 2.48), and SGA (aRR: 2.27, 95%CI: 1.90, 2.72), though the risk of preterm birth did not reach the statistical significance after adjusting for the covariates. CC only use was associated with the risk of preterm birth (aRR: 1.29, 95%CI: 1.18, 1.40), low birth weight (aRR: 1.78, 95%CI: 1.66, 1.91), and SGA (aRR: 2.02, 95%CI: 1.88, 2.17) in both unadjusted and adjusted analyses (Fig 2). Of the 8.0% women who had preterm birth infants, 6.2% (95%CI: 5.8%, 6.7%), 20.1% (95%CI: 19.3%, 20.9%) and 73.7% (95%CI: 72.8%, 74.6%) delivered <28 weeks, 28–33 weeks and 34–36 weeks, respectively. Similarly, among women who had low birth weight infants, 8.1% (95%CI: 7.6%, 8.7%), 9.4% (95%CI: 8.8%, 10.0%), and 82.5% (95%CI: 81.7%, 83.3%) had extreme low birth weight <1,000 grams, very low birth weight 1,000-<1,500 grams, and low birth weight 1,500-<2,500 grams, respectively (Table 2). Ordered logistic regression with categorical gestational age and birth weight showed consistent results (S1 Fig).

**Table 2:** Prevalence of preterm birth, low birth weight and SGA of women who gave live singleton births in 2016–2020 and participated in the PRAMS survey.

Outcomes	Non-use (N = 173,574)^a^		EC only use (N = 977)^a^		CC only use (N = 14,752) ^a^		Dual use (N = 1,404)^a^		Total (N = 190,707)^a^	
	No.^a^	% (95%CI)^b^	No.^a^	% (95%CI^b^	No.^a^	% (95%CI)^b^	No.^a^	% (95%CI)^b^	No.^a^	% (95%CI)^b^
Gestational age (weeks)										
Preterm birth	26,461	7.8 (7.6, 8.0)	206	10.4 (8.2, 13.1)	3,208	11.9 (11.2, 12.7)	324	10.0 (8.2, 12.3)	30,199	8.1 (7.9, 8.3)
<28	2,306	0.5 (0.4, 0.5)	26	1.0 (0.5, 1.9)	253	0.7 (0.6, 0.9)	26	0.5 (0.2, 1.2)	2,611	0.5 (0.5, 0.5)
28–33	7,280	1.5 (1.5, 1.6)	54	2.1 (1.4, 3.2)	803	2.1 (1.8, 2.3)	102	2.9 (2.0, 4.1)	8,239	1.6 (1.5, 1.6)
34–36	16,875	5.8 (5.6, 5.9)	126	7.3 (5.4, 9.8)	2,152	9.1 (8.4, 9.9)	196	6.7 (5.1, 8.6)	19,349	6.0 (5.9, 6.2)
Term birth	146,942	92.2 (92.0, 92.4)	770	89.6 (86.9, 91.8)	11,502	88.1 (87.3, 88.8)	1,077	90.0 (87.7, 91.8)	160,291	91.9 (91.7, 92.1)
Birth weight (grams)										
Low birth weight (<2,500)	30,482	6.1 (5.9, 6.2)	236	8.7 (7.0, 10.7)	4,532	12.2 (11.6, 12.8)	477	12.5 (10.4, 14.9)	35,727	6.5 (6.4, 6.6)
<1,000	2,326	0.5 (0.5, 0.5)	29	1.2 (0.7, 2.2)	266	0.7 (0.6, 0.9)	31	0.8 (0.4, 1.8)	2,652	0.5 (0.5, 0.5)
1,000-<1,500	2,833	0.6 (0.5, 0.6)	16	0.4 (0.2, 0.9)	306	0.8 (0.6, 1.0)	32	1.1 (0.5, 2.1)	3,187	0.6 (0.6, 0.6)
1,500-<2,500	25,323	5.0 (4.9, 5.1)	191	7.0 (5.6, 8.8)	3,960	10.7 (10.1, 11.3)	414	10.6 (8.8, 12.8)	29,888	5.4 (5.3, 5.5)
Normal birth weight (≥2,500)	142,899	93.9 (93.8, 94.1)	741	91.3 (89.3, 93.0)	10,206	87.8 (87.2, 88.4)	924	87.5 (85.1, 89.6)	154,770	93.5 (93.4, 93.6)
SGA										
Yes	22,751	8.9 (8.7, 9.1)	163	11.4 (8.7, 14.9)	3,689	19.0 (18.0, 20.0)	368	22.4 (19.0, 26.3)	26,971	9.7 (9.5, 9.9)
No	150,410	91.1 (90.9, 91.3)	812	88.6 (85.1, 91.3)	10,995	81.0 (80.0, 82.0)	1,031	77.6 (73.7, 81.0)	163,248	90.3 (90.1, 90.5)

^a^Unweighted sample size.

^b^Weighted prevalence and corresponding confidence interval (expressed as a percentage).

**Fig 3:**
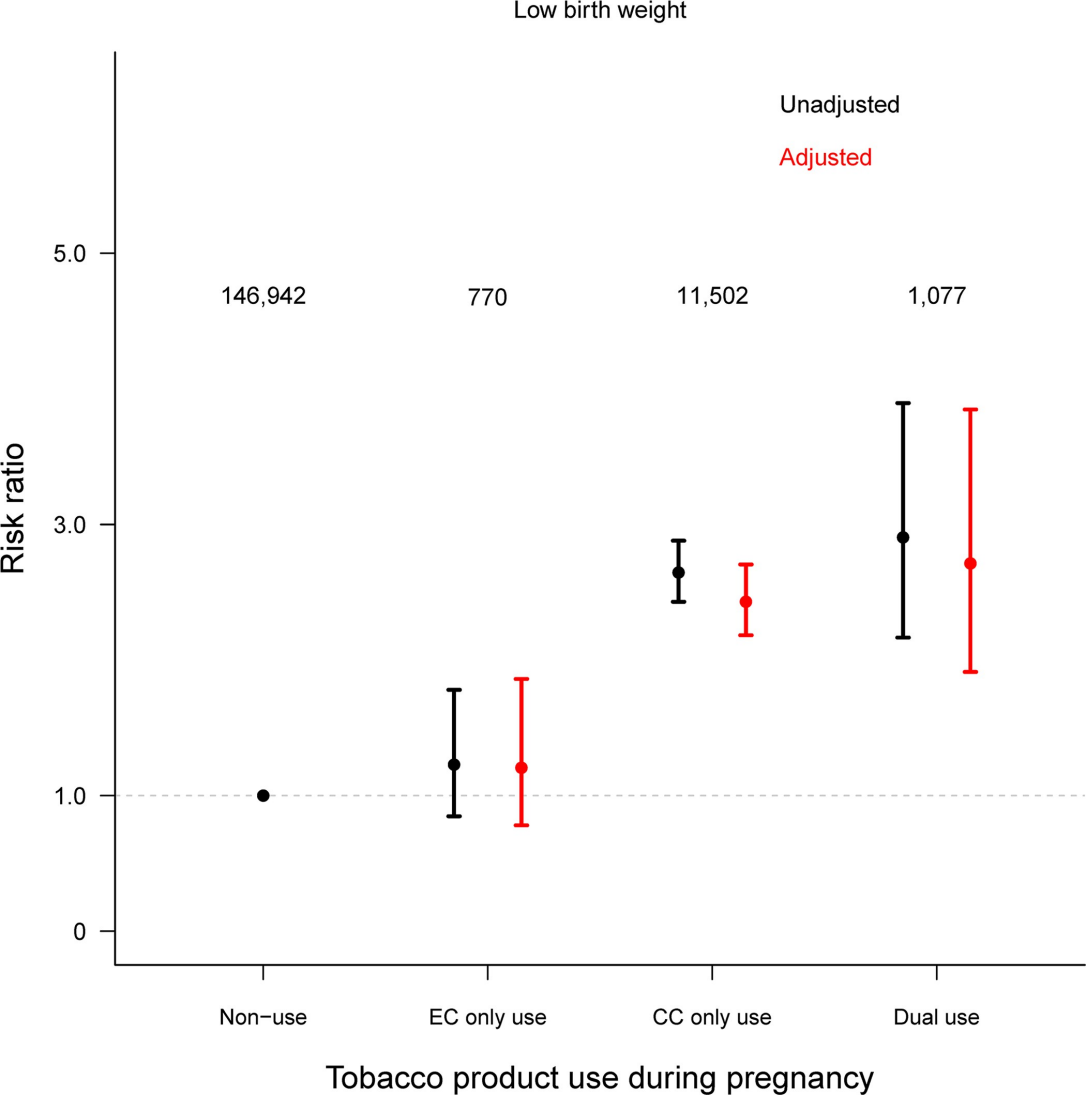
Risk of low birth weight comparing women of EC only use, CC only use, and dual use during pregnancy to women who were non-users in a subset of women who had had term birth babies (≥37 weeks). Numbers presented are unweighted sample sizes in each group. The multivariable modified Poisson regression (red) was adjusted for maternal age at delivery, maternal race/ethnicity, maternal education, marital status, household income, prenatal federal nutritional assistance, pregnancy intention, the Kotelchuck index, initiation of prenatal care in the first trimester, pre-pregnancy multivitamin use, pre-pregnancy alcoholic drinking frequency, parity, history of preterm birth, maternal pre-pregnancy BMI, delivery method, maternal residency, and year of delivery.

In the analysis exploring the association between tobacco products and low birth weight additionally adjusting for gestational age, the risk of low birth weight when comparing with non-use was decreased in all three tobacco product use groups (S2 Fig). While the risk remained statistically significant for CC only use (aRR: 1.54, 95%CI: 1.45, 1.64) and dual use (aRR: 1.71, 95%CI: 1.42, 2.06), the risk of low birth weight became non-significant for the EC only use group (aRR: 1.10, 95%CI: 0.90, 1.35). This non-statistically significant relationship between EC only use and low birth weight persisted in the subgroup of women who had had term birth infants (aRR: 1.21, 95%CI: 0.78, 1.86) (Fig 3). In contrast, dual use remained a significant risk factor for low birth weight (Fig 3).

To determine whether quitting EC use during pregnancy reduces the risk of adverse birth outcomes, we conducted the second analysis of 7,877 (weighted N = 422,533) women who self-reported EC use prior to pregnancy, with and without CC use (study population two). Among them, 75.3% (95%CI: 73.7%, 76.8%) quit EC use during pregnancy, and 24.7% (95%CI: 23.2%, 26.3%) continued EC use (Fig 1). Forty-three percent of women who used ECs prior to pregnancy were adolescents and/or young adults <25 years age group, and 76.7% were non-Hispanic White. Compared with women who continue using ECs during pregnancy, women who quit use were more likely to be race/ethnicities other than non-Hispanic White, have higher education, be married, have a higher household income, and be less likely to enroll in the federal food and nutritional assistance program (Table 3). Women who quit EC use were more likely to have an intended pregnancy, have at least “adequate” prenatal care, start prenatal care during the first trimester of pregnancy, use vitamins prior to becoming pregnant, be less likely to have live births prior to the current pregnancy and be less likely to have prior preterm birth (Table 3).

**Table 3:** Demographic, behavioral and health related characteristics of women EC users who gave live singleton births in 2016–2020 and participated in the PRAMS survey.

Characteristics	Continued use (n = 2,002^a^)		Quit use (n = 5,875^a^)		Overall	
	N^a^	% (95%CI)^b^	N	% (95%CI)	N	% (95%Cl)
Maternal age						
≤ 24	741	38.4 (34.9, 42.0)	2,661	44.6 (42.6, 46.7)	3,402	43.1 (41.3, 44.8)
25–29	599	28.6 (25.5, 31.9)	1,614	27.6 (25.8, 29.4)	2,213	27.8 (26.3, 29.4)
30–34	435	22.9 (20.1, 26.1)	1,057	18.0 (16.6, 19.6)	1,492	19.2 (17.9, 20.7)
≥35	226	10.0 (8.2, 12.3)	542	9.8 (8.6, 11.0)	768	9.8 (8.8, 10.9)
Maternal race/ethnicity						
Non Hispanic White	1,295	81.9 (79.4, 84.2)	3,449	74.9 (73.3, 76.6)	4,744	76.7 (75.3, 78.0)
Non Hispanic Black	178	4.9 (3.7, 6.4)	594	7.2 (6.3, 8.3)	772	6.7 (5.8, 7.5)
Hispanic	203	7.6 (6.1, 9.5)	802	10.7 (9.6, 12.0)	1,005	9.9 (9.0, 11.0)
Other	317	5.6 (4.5, 6.9)	991	7.1 (6.3, 8.0)	1,308	6.7 (6.0, 7.5)
Maternal education						
Some high school or less	421	20.7 (17.9, 24.0)	825	13.4 (12.0, 14.8)	1,246	15.2 (13.9, 16.5)
High school graduate	788	40.3 (36.8, 43.8)	2,087	37.0 (35.1, 39.1)	2,875	37.8 (36.1, 39.6)
Some college	642	31.7 (28.5, 35.1)	2,161	35.2 (33.3, 37.1)	2,803	34.3 (32.7, 36.0)
College graduate or more	133	7.3 (5.6, 9.4)	758	14.4 (13.1, 15.9)	891	12.7 (11.5, 13.9)
Marital status						
Married	615	32.2 (28.9, 35.6)	2,108	37.5 (35.5, 39.5)	2,723	36.2 (34.5, 37.9)
Other	1,376	67.8 (64.4, 71.1)	3,759	62.5 (60.5, 64.5)	5,135	63.8 (62.1, 65.5)
Household income ($)						
≤ 20,000	1,031	53.9 (50.3, 57.6)	2,333	40.2 (38.1, 42.2)	3,364	43.6 (41.8, 45.4)
20,001–40,000	478	22.3 (19.6, 25.4)	1,472	25.7 (23.9, 27.6)	1,950	24.9 (23.4, 26.5)
40,001–85,000	263	16.9 (14.2, 20.0)	1,124	22.2 (20.5, 24.0)	1,387	20.9 (19.4, 22.4)
≥85,001	101	6.8 (5.2, 8.8)	566	12.0 (10.7, 13.4)	667	10.7 (9.6, 11.8)
Maternal WIC use during pregnancy						
No	877	44.8 (41.2, 48.4)	3,009	55.0 (53.0, 57.1)	3,886	52.5 (50.7, 54.3)
Yes	1,090	55.2 (51.6, 58.8)	2,795	45.0 (42.9, 47.0)	3,885	47.5 (45.7, 49.3)
Pregnancy intention						
Unintended	697	36.4 (33.0, 39.9)	2,022	33.2 (31.3, 35.2)	2,719	34.0 (32.3, 35.7)
Intended	743	37.3 (33.9, 40.8)	2,423	44.1 (42.1, 46.1)	3,166	42.4 (40.7, 44.2)
Unsure	562	26.4 (23.3, 29.7)	1,430	22.7 (21.0, 24.4)	1,992	23.6 (22.1, 25.1)
Kotelcholck index						
Inadequate	412	18.3 (15.7, 21.3)	857	14.6 (13.2, 16.2)	1,269	15.5 (14.2, 16.9)
Intermediate	236	12.7 (10.3, 15.6)	609	10.6 (9.4, 11.9)	845	11.1 (10.0, 43.6)
Adequate	668	38.7 (35.2, 42.2)	2,309	42.8 (40.8, 44.9)	2,977	41.8 (40.1, 43.6)
Adequate Plus	626	30.3 (27.2, 33.7)	1,941	31.9 (30.1, 33.8)	2,567	31.5 (29.9, 33.2)
Prenatal care started in the 1^st^ trimester of pregnancy						
No	393	18.4 (15.9, 21.4)	797	13.4 (12.0, 15.0)	1,190	14.7 (13.4, 16.0)
Yes	1,506	79.2 (76.2, 82.0)	4,950	85.9 (84.3, 87.4)	6,456	84.3 (82.9, 85.6)
No Prenatal Visit	46	2.3 (1.3, 4.0)	40	0.7 (0.4, 1.1)	86	1.1 (0.7, 1.6)
Parity						
Primiparous	762	40.9 (37.4, 44.5)	3,216	55.0 (52.9, 57.0)	3,978	51.5 (49.7, 53.2)
2	564	27.3 (24.3, 30.4)	1,437	25.5 (23.8, 27.3)	2,001	25.9 (24.4, 27.5)
≥3	668	31.8 (28.6,35.2)	1,208	19.5 (18.0, 21.2)	1,876	22.6 (21.2, 24.1)
History of preterm birth						
No	1,859	95.4 (94.0, 96.5)	5,627	97.3 (96.6, 97.8)	7,486	96.8 (96.2, 97.3)
Yes	140	4.6 (3.5, 6.0)	236	2.7 (2.2, 3.4)	376	3.2 (2.7, 3.8)
Pre-pregnancy BMI (kg/m^2^)						
Underweight (<18.5 kg/m^2^)	124	6.0 (4.5, 7.9)	265	3.9 (3.3, 4.8)	389	4.5 (3.8, 5.2)
Normal (18.5–24.9 kg/m^2^)	791	39.0 (35.5, 42.6)	2,315	41.1 (39.1, 43.1)	3,106	40.6 (38.8, 42.3)
Overweight (25.0–29.9 kg/m^2^)	462	27.2 (24.1, 30.6)	1,443	24.9 (23.1, 26.7)	1,905	25.4 (23.9, 27.0)
Obese (30.0+ kg/m^2^)	564	27.8 (24.7, 31.2)	1,738	30.1 (28.2, 32.0)	2,302	29.5 (27.9, 31.2)
Pre-pregnancy multivitamin use per week						
Never	1,392	72.6 (69.4, 75.6)	4,030	69.6 (67.7, 71.4)	5,422	70.3 (68.7, 71.9)
1–3 times	157	6.7 (5.3, 8.5)	375	5.8 (5.0, 6.8)	532	6.0 (5.3, 6.9)
4–6 times	71	2.3 (1.6, 3.3)	244	4.1 (3.4, 4.9)	315	3.6 (3.1, 4.3)
Everyday	373	18.4 (15.8, 21.3)	1,207	20.6 (19.0, 22.2)	1,580	20.0 (18.7, 21.5)
Delivery method						
Vaginal	1,295	67.2 (63.8, 70.5)	3,802	66.1 (64.1, 68.0)	5,097	66.4 (64.7, 68.0)
Assisted	49	2.2 (1.4, 3.4)	211	3.8 (3.1, 4.7)	260	3.4 (2.8, 4.1)
C-section	657	30.6 (27.4, 34.0)	1,860	30.1 (28.3, 32.0)	2,517	30.2 (28.6, 31.9)
Average daily CC use before pregnancy						
No	515	27.9 (24.8, 31.3)	2,119	37.2 (35.2, 39.2)	2,634	34.9 (33.2, 36.6)
≤ 10	815	38.3 (34.8, 41.8)	2,602	43.2 (41.2, 45.2)	3,417	42.0 (40.2, 43.7)
11–20	447	23.7 (20.8, 26.9)	822	15.0 (13.6, 16.5)	1,269	17.1 (15.8, 18.5)
>20	199	10.1 (8.0, 12.5)	279	4.7 (3.9, 5.6)	478	6.0 (5.2, 6.9)
Average daily CC use during pregnancy						
No	834	46.1 (42.5, 49.7)	4,526	78.0 (76.2, 79.7)	5,360	70.1 (68.5, 71.7)
≤10	910	43.6 (40.1, 47.2)	1,148	19.0 (17.5, 20.7)	2,058	25.1 (23.6, 26.6)
11–20	173	7.8 (6.1, 9.9)	140	2.6 (2.0, 3.4)	313	3.9 (3.2, 4.6)
>20	61	2.5 (1.7, 3.7)	30	0.4 (0.2, 0.7)	91	0.9 (0.7, 1.3)
Year of delivery						
2016	333	15.9 (13.4, 18.7)	963	17.0 (15.5, 18.6)	1,296	16.7 (15.4, 18.1)
2017	320	16.9 (14.4, 19.6)	916	16.2 (14.8, 17.7)	1,236	16.4 (15.1, 17.7)
2018	421	20.2 (17.6, 23.1)	1,115	18.5 (17.0, 20.0)	1,536	18.9 (17.6, 20.2)
2019	458	22.5 (19.5, 25.7)	1,352	22.8 (21.2, 24.6)	1,810	22.7 (21.3, 24.3)
2020	470	24.6 (21.7, 27.8)	1,529	25.5 (23.8, 27.3)	1,999	25.3 (23.8, 26.8)

^a^Unweighted sample size.

^b^Weighted prevalence and corresponding confidence interval (expressed as a percentage).

Quitting EC use was associated with women’s frequency of pre-pregnancy EC use as well as the amount of pre-pregnancy and during-pregnancy CC use (if they dual use CCs). Women with greater frequency of EC use were less likely to quit ECs (less than one day per week [79.1%] vs. 2–6 days per week [75.4%] vs. once per day [74.1%] vs. more than once per day [71.8%]). Among those who continued using ECs during pregnancy, the majority maintained their frequency of use (Table 4). Similarly, women who were never or light users of CCs before pregnancy and/or during pregnancy were more likely to quit EC use during pregnancy (Table 3).

**Table 4:** Change in the frequency of EC use during pregnancy among women EC users who gave live singleton births in 2016–2020 and participated in the PRAMS survey.

Frequency of EC use during pregnancy	Frequency of EC use before pregnancy								Total	
	<1 day per week		2–6 days per week		Once per day		>1 per day			
	N^a^	% (95%CI)^b^	N^a^	% (95%CI)^b^	N^a^	% (95%CI)^b^	N^a^	% (95%CI)^b^	N^a^	% (95%CI)^b^
No use^c^	2,415	79.1 (76.6, 81.4)	774	75.4 (71.1, 79.3)	566	74.1 (68.9, 78.8)	2,120	71.8 (69.2, 74.3)	5,875	75.3 (73.7, 76.8)
<1 day per week	537	17.6 (15.5, 20.0)	88	6.7 (4.9, 9.1)	31	3.6 (2.0, 6.4)	91	2.8 (2.0, 4.0)	747	9.0 (8.0, 10.1)
2–6 days per week	52	1.7 (1.2, 2.6)	142	13.5 (10.5, 17.2)	13	1.9 (0.9, 4.1)	73	1.7 (1.1, 2.5)	280	3.2 (2.7, 3.9)
Once per day	27	0.8 (0.4, 1.7)	29	2.2 (1.2, 4.1)	122	15.3 (11.7, 19.9)	111	3.6 (2.6, 4.9)	289	3.5 (2.9, 4.2)
>1 per day	22	0.8 (0.4, 1.6)	29	2.1 (1.1, 4.0)	32	5.0 (3.0, 8.1)	603	20.1 (17.9, 22.4)	686	9.0 (8.1, 10.1)

^a^Unweighted sample size.

^b^Weighted prevalence and corresponding confidence interval (expressed as a percentage).

^c^People who quit EC use during pregnancy

**Fig 4:**
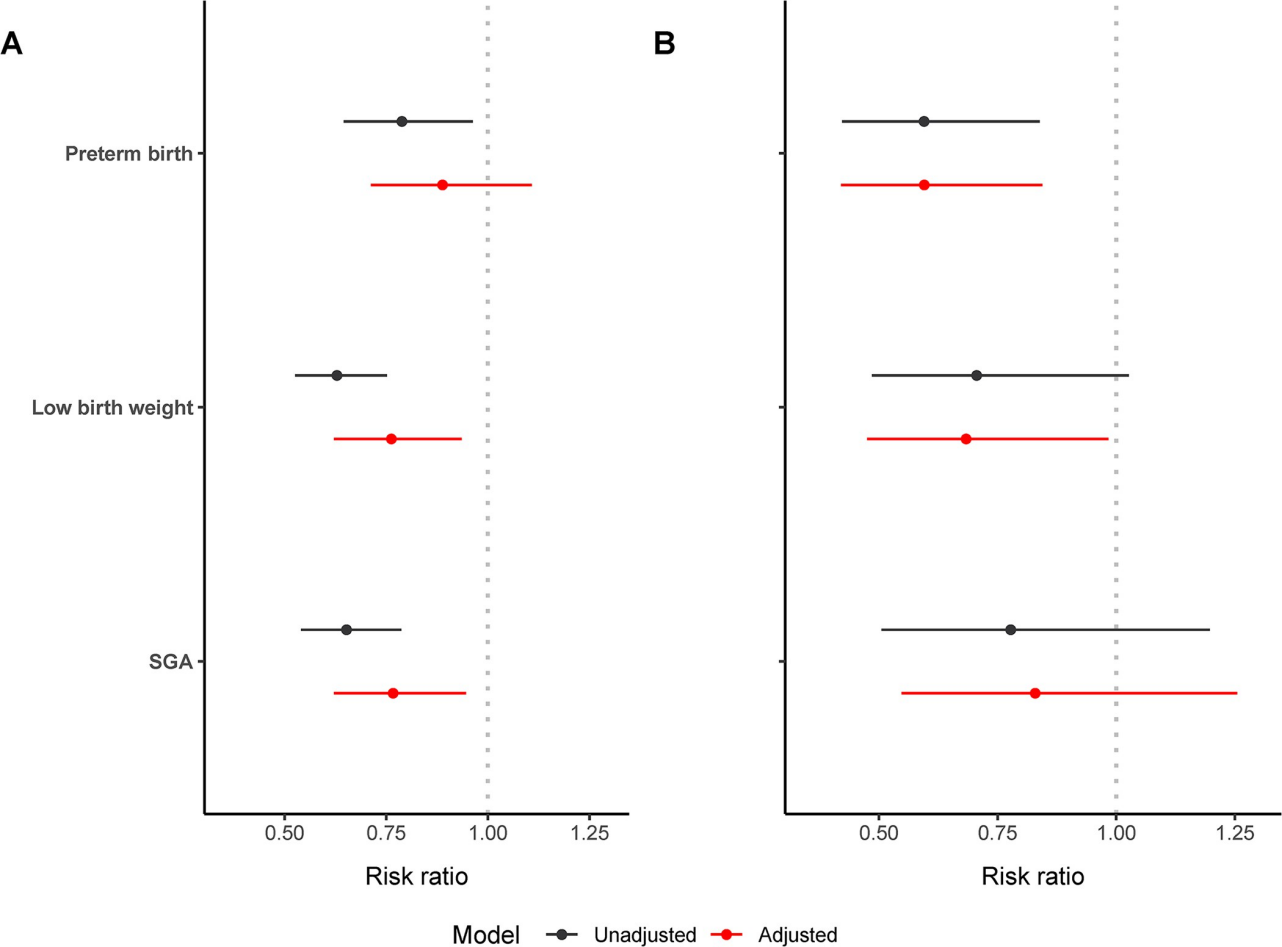
Risk of preterm birth, low birth weight and SGA comparing women who quit EC use during pregnancy to those who continued EC use in the population of women. (A) who all used EC prior to pregnancy and (B) the subset of EC only users (no use of CCs both before and during pregnancy). The multivariable modified Poisson regression was adjusted for maternal age at delivery, maternal race/ethnicity, maternal education, marital status, household income, prenatal federal nutritional assistance, pregnancy intention, the Kotelchuck index, initiation of prenatal care in the first trimester, pre-pregnancy multivitamin use, pre-pregnancy alcoholic drinking frequency, parity, history of preterm birth, maternal pre-pregnancy BMI, delivery method, maternal residency, and year of delivery. Number of CCs used before and during pregnancy was additionally adjusted in the overall population of women EC users (A).

Quitting EC use was associated with a significantly reduced risk of low birth weight (aRR: 0.76, 95%CI: 0.62, 0.94) and SGA (aRR: 0.77, 95%CI: 0.62, 0.94), in both univariate and adjusted analyses. The significantly reduced risk of preterm birth became non-significant after adjusting for covariates (aRR: 0.89, 95%CI: 0.71, 1.11) (Fig 4). In the subset of 34.6% women who did not use CCs both before and during pregnancy, quitting EC use was associated with a significantly reduced risk of preterm birth (aRR: 0.68, 95%CI: 0.48, 0.98) and low birth weight (aRR: 0.60, 95%CI: 0.42, 0.84), but not SGA (aRR: 0.81, 95%CI: 0.54, 1.23) (Fig 4).

There were 32,519 (17.1%) and 1,223 (15.5%) subjects missing one or more covariates in the two study populations respectively (S3 and S4 Tables). Household income was the most common variable missing, with a weighted 8.63% and 6.74% subjects having missing household income respectively in the two study populations. Multiple imputation analyses showed consistent results (S3–S7 Figs).

## Discussion

Our large surveillance study of pregnant women who had live singleton births in 2016–2020 yielded several important findings regarding EC use during pregnancy that could inform public health policy. EC use during the last three months of pregnancy is not common among pregnant individuals. Of the 1.2% women who self-reported using ECs during the last three months of pregnancy, 43.9% used ECs only and 56.1% dual used ECs and CCs. The low EC use is partially because the majority of the women EC users quit EC use when they become pregnant. EC only use is associated with an increased risk of preterm birth and low birth weight, but not SGA. Dual use is associated with an increased risk of preterm birth, low birth weight, and SGA. The association between EC only use and low birth weight becomes non-significant when additionally adjusting for gestational age and in the term birth population, while the risk of dual use on these birth outcomes remains significant. At the same time, quitting EC use during pregnancy is associated with a significantly reduced risk of these adverse birth outcomes.

Our results confirm and expand upon previous reports of the association between pregnancy EC use and poor birth outcomes and provide an explanation for inconsistencies in findings among prior studies [24–30]. Animal studies have shown that prenatal EC exposure impacts fetal growth. Chronic exposure to EC aerosols containing nicotine during pregnancy has deleterious health effects on the offspring, leading to reduction in offspring weight and crown-rump length at birth and days after birth [31]. In humans, studies have reported inconsistent results ranging from a significantly increased risk to no association with preterm birth, low birth weight, and/or SGA [24–30]. Our results suggest a possible explanation for the previously reported inconsistent findings. Specifically, we found that EC only use is associated with preterm birth and it is potentially through increasing the risk of preterm birth that we see an increase in the risk of low birth weight, as the association with low birth weight is no longer apparent after adjustment for gestational age or when analysis is restricted to term births. Further, we observed no association between EC only use and SGA, a measure representing children’s birth weight relative to their gestational age. Similar findings were reported in a prospective cohort of 620 live singleton births in Ireland [29]. When all infants were term births, EC only use, determined in the 10–14 weeks of pregnancy, was not associated with birth weight or SGA compared to non-use. The potential harm of EC only use during pregnancy on adverse birth outcomes is reinforced by our result of a reduced risk found by comparing women who quit use with those who continued its use.

Our finding of a significantly increased risk of dual use of ECs and CCs on the risk of preterm birth, low birth weight, and SGA is consistent with prior scholarship [24, 26, 29, 30]. Women dual users have a similar risk compared to the CC only users who are well-established to have an increased risk of adverse birth outcomes, suggesting that CCs may be a unique and profound driver of adverse birth outcomes relative to ECs, directly affecting intrauterine fetal growth.

Biological mechanisms for adverse pregnancy-related outcomes following prenatal EC use are relatively unknown, as are the mechanisms driving the difference in findings between ECs and CCs. E-liquids and the products (e.g., aldehydes) formed during the heating process can both contribute to the increased risk [32]. Animal studies have shown that exposure to EC aerosols containing nicotine during pregnancy reduces blood flow in both the maternal uterine artery and fetal umbilical cord, which could result in both intrauterine growth restriction and preterm birth [33]. Ingredients other than nicotine have also been shown to hinder the function of trophoblasts in the placenta and lead to complications in placental structuring [17, 18]. Such placental changes could contribute to later increased risk of preeclampsia, early miscarriage, premature birth and even maternal or fetal death, but do not fully explain the differences in effect of ECs compared with CCs.

A unique contribution is the finding that quitting EC use during pregnancy is associated with reduced risk of adverse birth outcomes, a result consistent with the beneficial effect of quitting smoking. To date, ECs are classified as a tobacco product and no ECs have been approved as a smoking cessation aid in the pregnant women population. A recent Cochrane review concludes that nicotine-containing ECs are effective for smoking cessation [16]. Our finding that ECs may be harmful during pregnancy and quitting reduces the harm, in combination with prior scholarship, reinforce current public health advising against the use of ECs during pregnancy. Pregnant women smokers should be cautious when considering ECs as a viable smoking cessation strategy, and those EC users should be encouraged to quit EC use.

The strengths of this study includes the large study population of the PRAMS data which represents approximately 81% of live births in the United States and across five years of births [19]. PRAMS data are linked to the birth certificate, allowing the extraction of outcomes and covariates of interest which have been demonstrated to be accurate in previous studies [34, 35]. Despite these strengths, this study also has several limitations inherent to most retrospective secondary data analyses. First, our study is limited to women with live births. EC use may affect fetal survival and thus bias the association towards the null. Second, we relied on self-reported EC and CC use in the last three months of pregnancy, which as past studies suggest, is fairly accurate and representative of an individual’s smoking pattern in pregnancy [36–39]. We assumed that EC users quit using ECs during pregnancy if they reported no use in the last three months of pregnancy. It is plausible the women EC users continued using ECs early in pregnancy although such misclassification will drive the quitting effect toward the null. Third, the PRAMS questionnaire aggregated usage of multiple types of e-cigarette products. It is thus not possible to differentiate specific effect of different types of ECs. Studies have shown that types of device (pods vs tanks, coil type, device power output setting) and choice of e-liquid (nicotine-containing versus nicotine-free e-cigarettes, flavoring agents and chemicals) affect a user’s vaping topography and the amount of nicotine and other chemicals a person inhales [40–42]. Some devices therefore have higher addiction potential and thus, may be more difficult to quit using. Most pod type devices contain nicotine in salt form that may be more potent and thus, affect birth outcomes. Future studies that collect device information and directly measure nicotine and chemicals pregnant women inhale will help address product specific effect of ECs on preterm birth and low birth weight. We determined preterm birth based on a woman’s gestational age regardless of the type of preterm birth. We could not differentiate spontaneous preterm birth from medically indicated preterm birth of which preeclampsia is the leading cause [43]. It is plausible that the effect of EC use on preterm birth differs by the type of preterm birth; however, this could not be differentiated in this study. Lastly, given the observational and cross-sectional nature of the PRAMS survey, causal conclusions between EC use and adverse outcomes cannot be ascertained. Future studies that prospectively follow women regarding their pregnancy EC use and pregnancy outcomes may allow the establishment of causal inference.

## Conclusion

In this large surveillance survey study of U.S. pregnant women, EC use in pregnancy, by itself or dual use with CC, is associated with an increased risk of preterm birth. Through increasing the risk of preterm birth, EC use increases the risk of low birth weight. Quitting EC use reduces the risk. Pregnant women or those planning to become pregnant who smoke and consider ECs as a healthier alternative and/or as a smoking cessation tool should take into account the potential adverse effect of ECs. Pregnant EC users should be advised for quitting EC use.

## Supporting information

S1 Checklist STROBE statement—checklist of items that should be included in reports of *cross-sectional studies*. (DOC)

S1 Fig Odds of (A) preterm birth and (B) low birth weight as categorical variables comparing women EC only use, CC only use, and dual use during pregnancy to women who were non-users. Numbers presented are unweighted sample sizes in each group. The multivariable proportional odds ratio model (red) was adjusted for maternal age at delivery, maternal race/ethnicity, maternal education, marital status, household income, prenatal federal nutritional assistance, pregnancy intention, the Kotelchuck index, initiation of prenatal care in the first trimester, pre-pregnancy multivitamin use, pre-pregnancy alcoholic drinking frequency, parity, history of preterm birth, maternal pre-pregnancy BMI, maternal residency, and year of delivery.
(TIF)

S2 Fig Risk of low birth weight comparing women of EC only use, CC only use, and dual use during pregnancy to women who were non-users. Numbers presented are unweighted sample sizes in each group. The multivariable modified Poisson regression (red) was adjusted for maternal age at delivery, maternal race/ethnicity, maternal education, marital status, household income, prenatal federal nutritional assistance, pregnancy intention, the Kotelchuck index, initiation of prenatal care in the first trimester, pre-pregnancy multivitamin use, pre-pregnancy alcoholic drinking frequency, parity, history of preterm birth, maternal pre-pregnancy BMI, maternal residency, and year of delivery. A second multivariable model was additionally adjusted for gestational age (purple).
(TIF)

S3 Fig Risk of (A) preterm birth, (B) low birth weight, and (C) SGA comparing women of EC only use, CC only use, and dual use during pregnancy to women who were non-users. Numbers presented are unweighted sample sizes in each group. The multivariable modified Poisson regression (red) was performed with multiple imputation for missing covariates. The covariates included maternal age at delivery, maternal race/ethnicity, maternal education, marital status, household income, prenatal federal nutritional assistance, pregnancy intention, the Kotelchuck index, initiation of prenatal care in the first trimester, pre-pregnancy multivitamin use, pre-pregnancy alcoholic drinking frequency, parity, history of preterm birth, maternal pre-pregnancy BMI, maternal residency, and year of delivery.
(TIF)

S4 Fig Odds of (A) preterm birth and (B) low birth weight as categorical variables comparing women EC only use, CC only use, and dual use during pregnancy to women who were non-users. Numbers presented are unweighted sample sizes in each group. The multivariable proportional odds ratio model (red) was performed with multiple imputation for missing covariates. The covariates included maternal age at delivery, maternal race/ethnicity, maternal education, marital status, household income, prenatal federal nutritional assistance, pregnancy intention, the Kotelchuck index, initiation of prenatal care in the first trimester, pre-pregnancy multivitamin use, pre-pregnancy alcoholic drinking frequency, parity, history of preterm birth, maternal pre-pregnancy BMI, maternal residency, and year of delivery.
(TIF)

S5 Fig Risk of low birth weight comparing women of EC only use, CC only use, and dual use during pregnancy to women who were non-users. Numbers presented are unweighted sample sizes in each group. The multivariable modified Poisson regression (red) was performed with multiple imputation for missing covariates. The covariates included maternal age at delivery, maternal race/ethnicity, maternal education, marital status, household income, prenatal federal nutritional assistance, pregnancy intention, the Kotelchuck index, initiation of prenatal care in the first trimester, pre-pregnancy multivitamin use, pre-pregnancy alcoholic drinking frequency, parity, history of preterm birth, maternal pre-pregnancy BMI, maternal residency, and year of delivery. A second multivariable model additionally adjusted for gestational age (purple).
(TIF)

S6 Fig Risk of low birth weight comparing women of EC only use, CC only use, and dual use during pregnancy to women who were non-users in a subset of women who had had term birth babies (≥37 weeks). Numbers presented are unweighted sample sizes in each group. The multivariable modified Poisson regression (red) was performed with multiple imputation for missing covariates. The covariates included maternal age at delivery, maternal race/ethnicity, maternal education, marital status, household income, prenatal federal nutritional assistance, pregnancy intention, the Kotelchuck index, initiation of prenatal care in the first trimester, pre-pregnancy multivitamin use, pre-pregnancy alcoholic drinking frequency, parity, history of preterm birth, maternal pre-pregnancy BMI, delivery method, maternal residency, and year of delivery.
(TIF)

S7 Fig Risk of preterm birth, low birth weight and SGA comparing women who quit EC use during pregnancy to those who continued EC use in the population of women (A) who all used EC prior to pregnancy and (B) the subset of EC only users (no use of CCs both before and during pregnancy). The multivariable modified Poisson regression was performed with multiple imputation for missing covariates. The covariates included maternal age at delivery, maternal race/ethnicity, maternal education, marital status, household income, prenatal federal nutritional assistance, pregnancy intention, the Kotelchuck index, initiation of prenatal care in the first trimester, pre-pregnancy multivitamin use, pre-pregnancy alcoholic drinking frequency, parity, history of preterm birth, maternal pre-pregnancy BMI, delivery method, maternal residency, and year of delivery. Number of CCs used before and during pregnancy was additionally adjusted in the overall population of women EC users (A).
(TIF)

S1 Table Participating sites meeting minimum response rate thresholds and providing data for this study by year, PRAMS, 2016–2020. (DOCX)

S2 Table Measurement of cigarette use in the PRAMS Survey, 2016–2020. (DOCX)

S3 Table Frequency and proportion of subjects missing in one or more covariates in the population of women who gave live singleton births in 2016–2020, PRAMS. (DOCX)

S4 Table Frequency and proportion of subjects missing in one or more covariates in the population of women who all used ECs prior to pregnancy and gave live singleton births in 2016–2020, PRAMS. (DOCX)
