# Supplementary figures and images for: Electronic cigarette use during pregnancy and the risk of adverse birth outcomes: A cross-sectional surveillance study of the US Pregnancy Risk Assessment Monitoring System (PRAMS) population
Source: PLoS One. 2023 Oct 24;18(10):e0287348. doi: 10.1371/journal.pone.0287348 (PMC10597477; doi:10.1371/journal.pone.0287348)

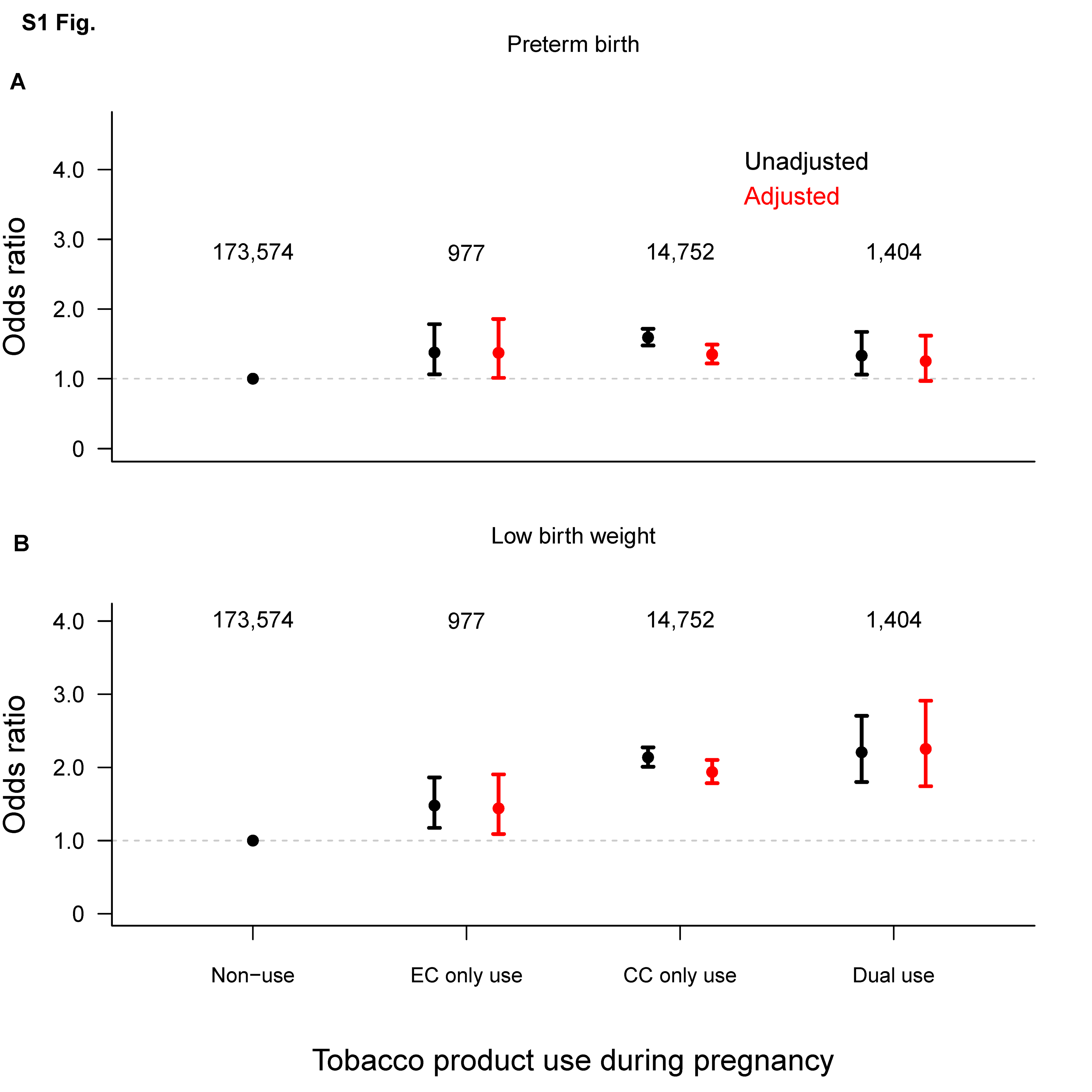

Supplement: S1 Fig — Odds of (A) preterm birth and (B) low birth weight as categorical variables comparing women EC only use, CC only use, and dual use during pregnancy to women who were non-users. Numbers presented are unweighted sample sizes in each group. The multivariable proportional odds ratio model (red) was adjusted for maternal age at delivery, maternal race/ethnicity, maternal education, marital status, household income, prenatal federal nutritional assistance, pregnancy intention, the Kotelchuck index, initiation of prenatal care in the first trimester, pre-pregnancy multivitamin use, pre-pregnancy alcoholic drinking frequency, parity, history of preterm birth, maternal pre-pregnancy BMI, maternal residency, and year of delivery. (TIF) [file pone.0287348.s002.tif]

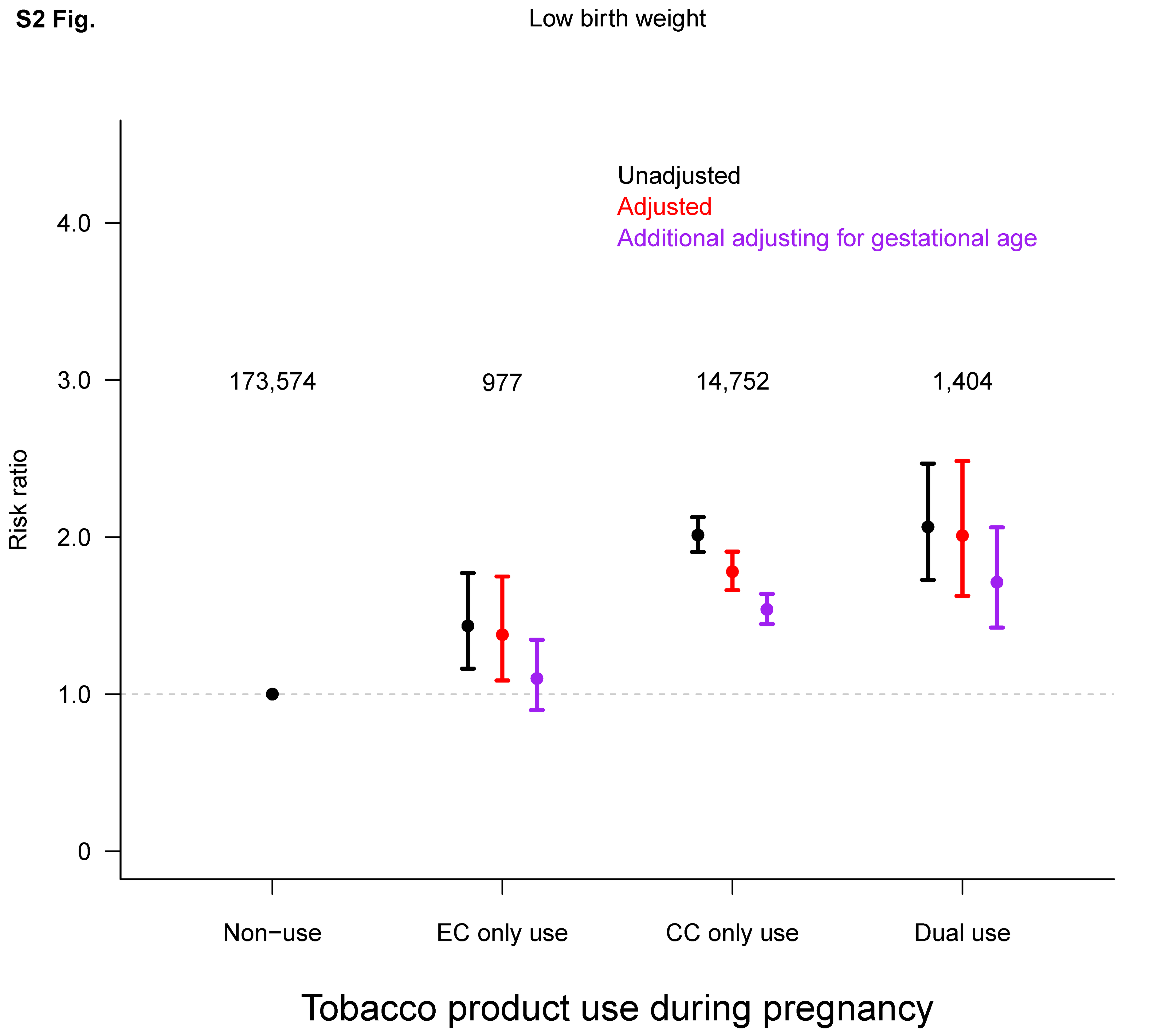

Supplement: S2 Fig — Numbers presented are unweighted sample sizes in each group. The multivariable modified Poisson regression (red) was adjusted for maternal age at delivery, maternal race/ethnicity, maternal education, marital status, household income, prenatal federal nutritional assistance, pregnancy intention, the Kotelchuck index, initiation of prenatal care in the first trimester, pre-pregnancy multivitamin use, pre-pregnancy alcoholic drinking frequency, parity, history of preterm birth, maternal pre-pregnancy BMI, maternal residency, and year of delivery. A second multivariable model was additionally adjusted for gestational age (purple). (TIF) [file pone.0287348.s003.tif]

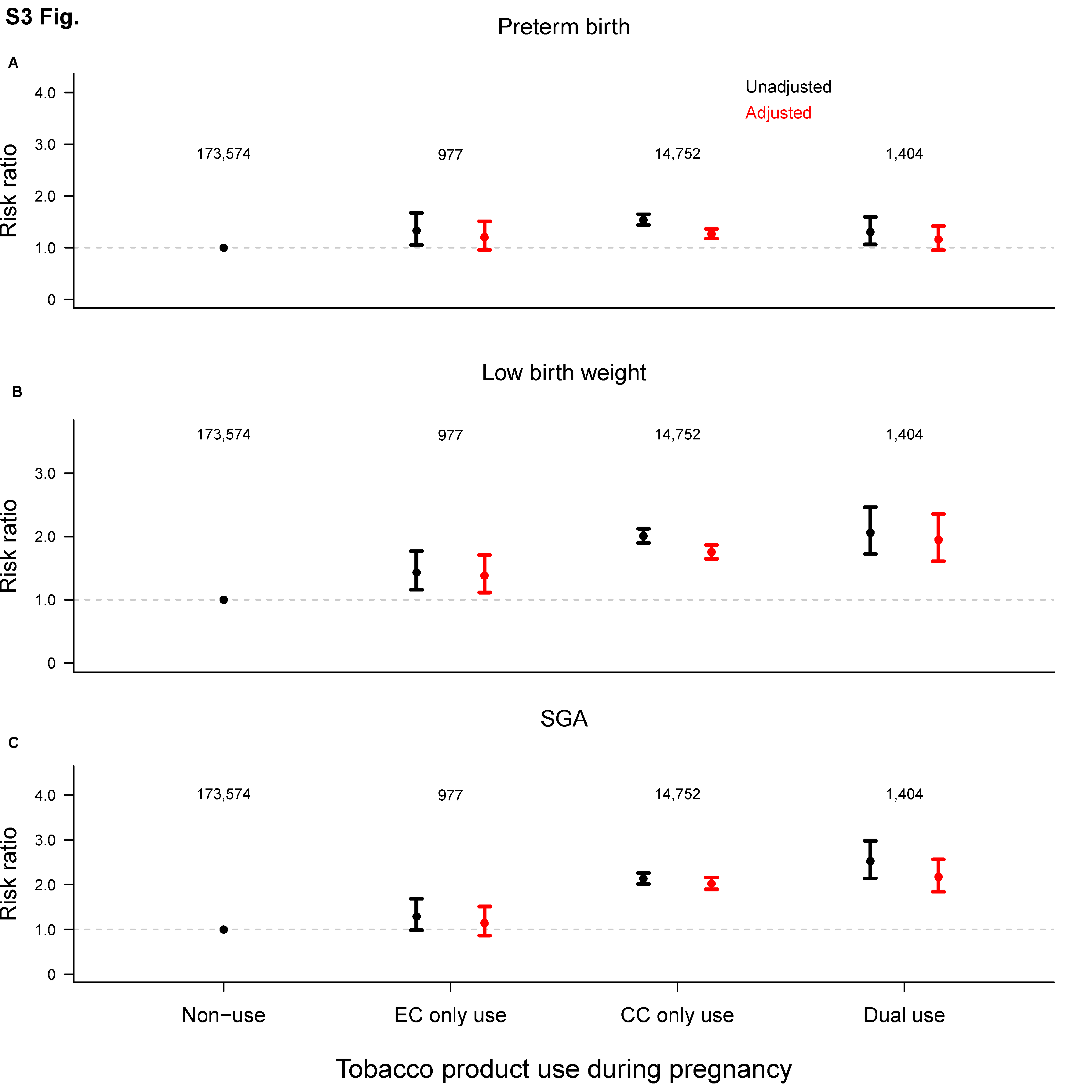

Supplement: S3 Fig — Risk of (A) preterm birth, (B) low birth weight, and (C) SGA comparing women of EC only use, CC only use, and dual use during pregnancy to women who were non-users. Numbers presented are unweighted sample sizes in each group. The multivariable modified Poisson regression (red) was performed with multiple imputation for missing covariates. The covariates included maternal age at delivery, maternal race/ethnicity, maternal education, marital status, household income, prenatal federal nutritional assistance, pregnancy intention, the Kotelchuck index, initiation of prenatal care in the first trimester, pre-pregnancy multivitamin use, pre-pregnancy alcoholic drinking frequency, parity, history of preterm birth, maternal pre-pregnancy BMI, maternal residency, and year of delivery. (TIF) [file pone.0287348.s004.tif]

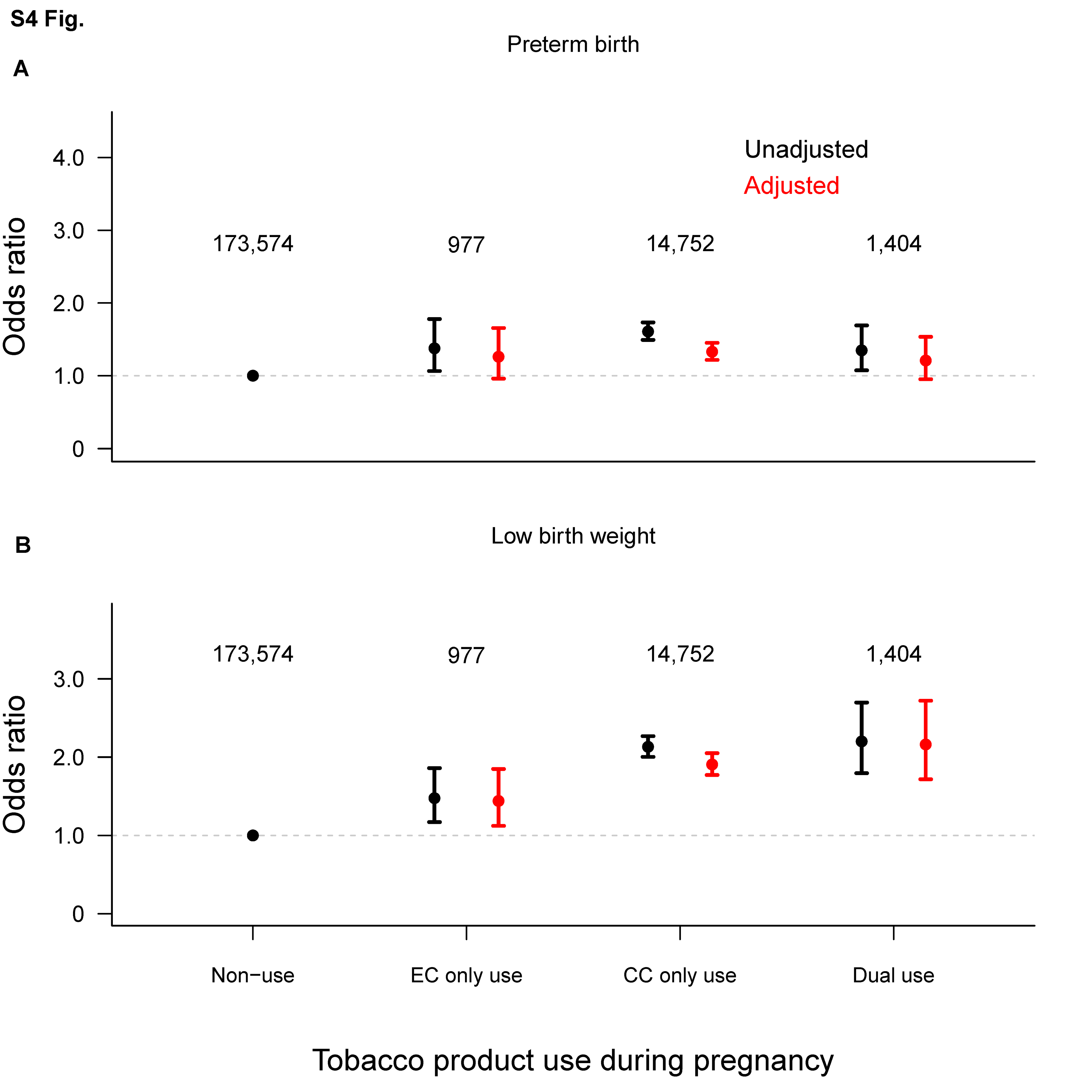

Supplement: S4 Fig — Odds of (A) preterm birth and (B) low birth weight as categorical variables comparing women EC only use, CC only use, and dual use during pregnancy to women who were non-users. Numbers presented are unweighted sample sizes in each group. The multivariable proportional odds ratio model (red) was performed with multiple imputation for missing covariates. The covariates included maternal age at delivery, maternal race/ethnicity, maternal education, marital status, household income, prenatal federal nutritional assistance, pregnancy intention, the Kotelchuck index, initiation of prenatal care in the first trimester, pre-pregnancy multivitamin use, pre-pregnancy alcoholic drinking frequency, parity, history of preterm birth, maternal pre-pregnancy BMI, maternal residency, and year of delivery. (TIF) [file pone.0287348.s005.tif]

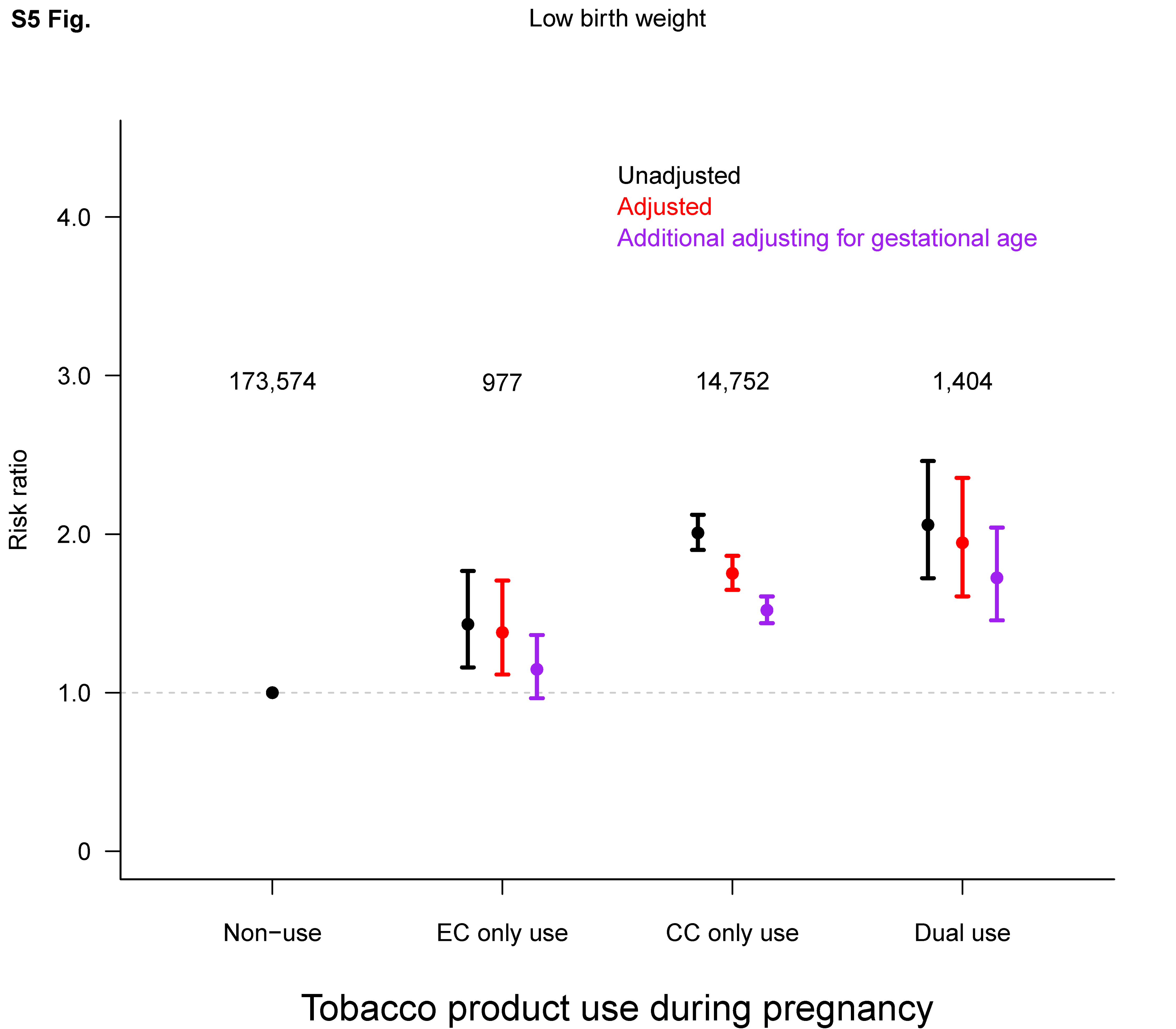

Supplement: S5 Fig — Numbers presented are unweighted sample sizes in each group. The multivariable modified Poisson regression (red) was performed with multiple imputation for missing covariates. The covariates included maternal age at delivery, maternal race/ethnicity, maternal education, marital status, household income, prenatal federal nutritional assistance, pregnancy intention, the Kotelchuck index, initiation of prenatal care in the first trimester, pre-pregnancy multivitamin use, pre-pregnancy alcoholic drinking frequency, parity, history of preterm birth, maternal pre-pregnancy BMI, maternal residency, and year of delivery. A second multivariable model additionally adjusted for gestational age (purple). (TIF) [file pone.0287348.s006.tif]

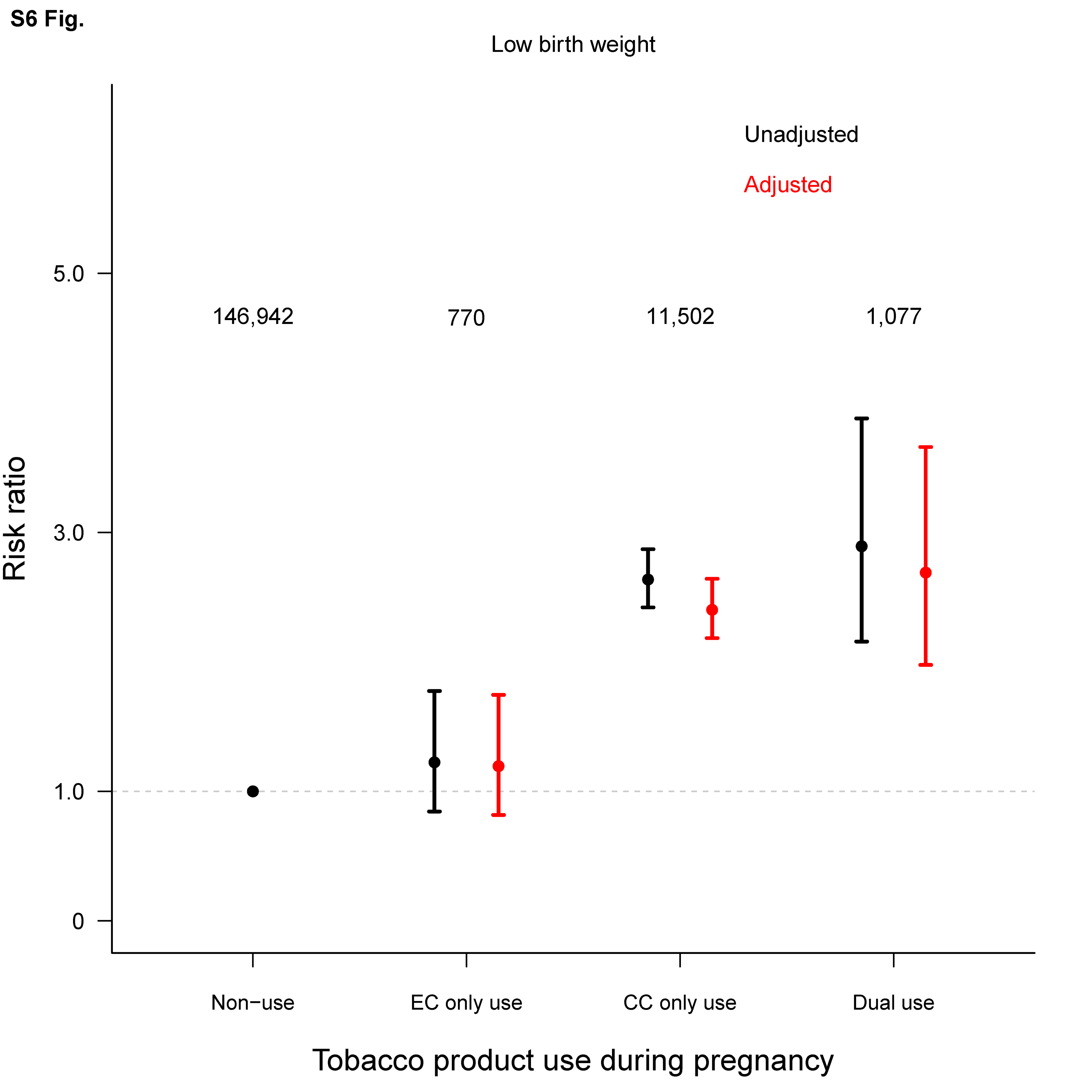

Supplement: S6 Fig — Numbers presented are unweighted sample sizes in each group. The multivariable modified Poisson regression (red) was performed with multiple imputation for missing covariates. The covariates included maternal age at delivery, maternal race/ethnicity, maternal education, marital status, household income, prenatal federal nutritional assistance, pregnancy intention, the Kotelchuck index, initiation of prenatal care in the first trimester, pre-pregnancy multivitamin use, pre-pregnancy alcoholic drinking frequency, parity, history of preterm birth, maternal pre-pregnancy BMI, delivery method, maternal residency, and year of delivery. (TIF) [file pone.0287348.s007.tif]

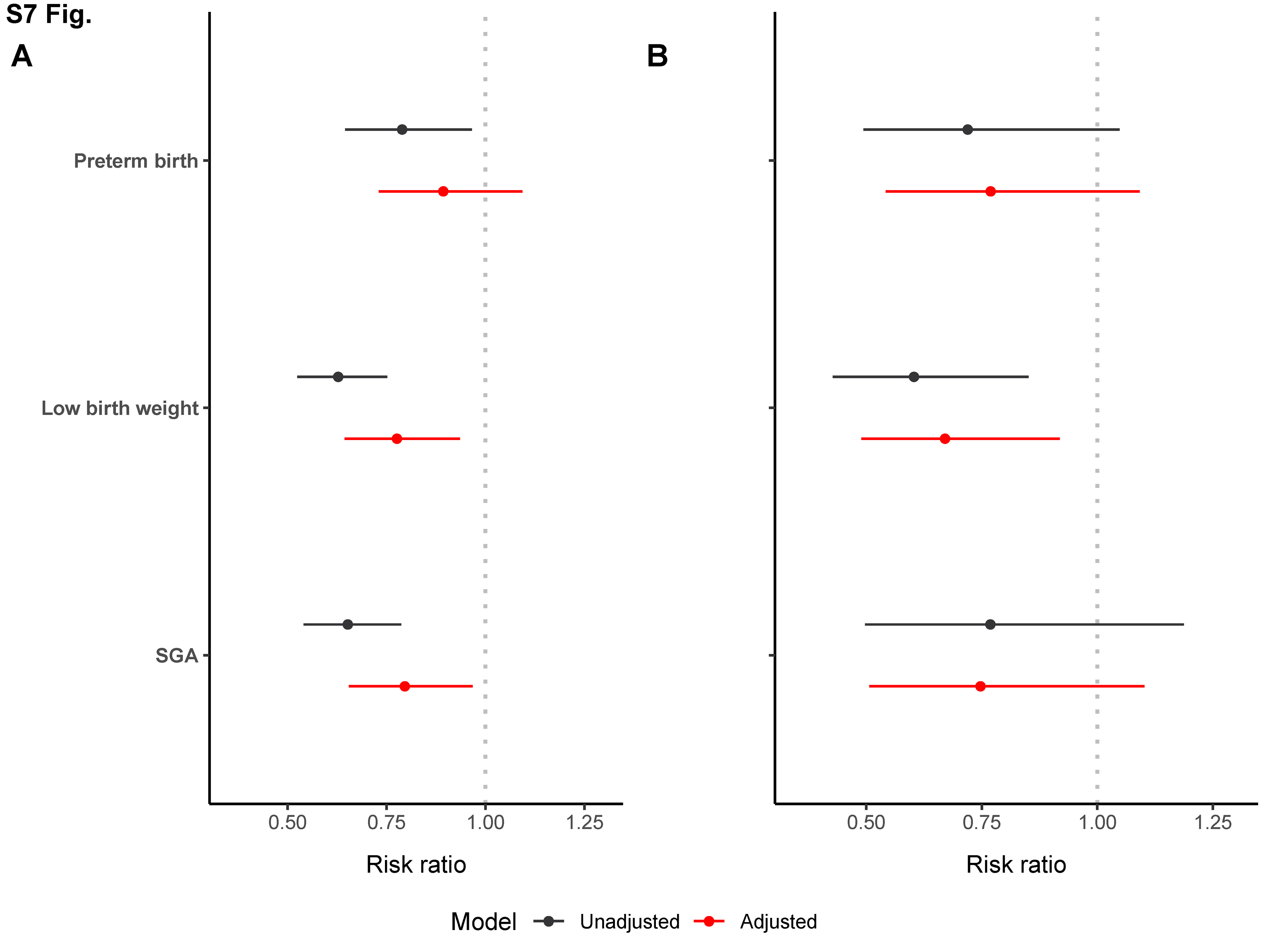

Supplement: S7 Fig — Risk of preterm birth, low birth weight and SGA comparing women who quit EC use during pregnancy to those who continued EC use in the population of women (A) who all used EC prior to pregnancy and (B) the subset of EC only users (no use of CCs both before and during pregnancy). The multivariable modified Poisson regression was performed with multiple imputation for missing covariates. The covariates included maternal age at delivery, maternal race/ethnicity, maternal education, marital status, household income, prenatal federal nutritional assistance, pregnancy intention, the Kotelchuck index, initiation of prenatal care in the first trimester, pre-pregnancy multivitamin use, pre-pregnancy alcoholic drinking frequency, parity, history of preterm birth, maternal pre-pregnancy BMI, delivery method, maternal residency, and year of delivery. Number of CCs used before and during pregnancy was additionally adjusted in the overall population of women EC users (A). (TIF) [file pone.0287348.s008.tif]
