# Supplementary material for: Electronic cigarette use during pregnancy and the risk of adverse birth outcomes: A cross-sectional surveillance study of the US Pregnancy Risk Assessment Monitoring System (PRAMS) population
Source: PLoS One. 2023 Oct 24;18(10):e0287348. doi: 10.1371/journal.pone.0287348 (PMC10597477; doi:10.1371/journal.pone.0287348)
Supplement: S1 Table — (DOCX) [file pone.0287348.s009.docx]

**S1 Table.** **Participating sites meeting minimum response rate thresholds and providing data for this study by year, PRAMS, 2016-2020.**

| Site | Study year | | | | |
| --- | --- | --- | --- | --- | --- |
|  | 2016^a^ | 2017^a^ | 2018^b^ | 2019^b^ | 2020^b^ |
| Alabama |  | x | x | x | x |
| Alaska | x | x | x | x | x |
| Arkansas | x |  | x | x | x |
| Arizona |  |  |  |  | x |
| Colorado | x | x | x | x | x |
| Connecticut | x | x | x | x | x |
| District of Columbia |  |  | x | x | x |
| Delaware | x | x | x | x | x |
| Florida |  |  |  | x | x |
| Georgia |  | x | x | x | x |
| Hawaii | x |  |  | x | x |
| Iowa | x | x | x | x | x |
| Illinois | x | x | x | x | x |
| Indiana |  |  | x |  |  |
| Kansas |  | x | x | x | x |
| Kentucky |  | x | x | x | x |
| Louisiana | x | x | x | x | x |
| Massachusetts | x | x | x | x | x |
| Maryland | x | x | x | x | x |
| Maine | x | x | x | x | x |
| Michigan | x | x | x | x | x |
| Minnesota |  |  | x | x | x |
| Missouri | x | x | x | x | x |
| Mississippi |  |  | x | x | x |
| Montana |  | x | x | x | x |
| North Carolina |  | x | x | x |  |
| North Dakota |  | x | x | x | x |
| Nebraska | x |  | x | x | x |
| New Hampshire | x | x | x | x | x |
| New Jersey | x | x | x | x | x |
| New Mexico | x | x | x | x | x |
| New York | x | x | x | x |  |
| Oklahoma | x | x | x |  |  |
| Oregon |  |  | x | x | x |
| Pennsylvania | x | x | x | x | x |
| Puerto Rico |  | x | x | x | x |
| Rhode Island | x | x | x | x |  |
| South Dakota |  | x | x | x | x |
| Tennessee |  |  |  | x | x |
| Texas | x |  |  |  |  |
| Utah | x | x | x | x | x |
| Virginia | x | x | x | x | x |
| Vermont | x | x | x | x | x |
| Washington | x | x | x | x | x |
| Wisconsin | x | x | x | x | x |
| West Virginia | x | x | x |  | x |
| Wyoming | x | x | x | x | x |
| New York City | x | x | x | x | x |
| ^a^55% response rate threshold for data to be available for analysis. | | | | | |
| ^b^50% response rate threshold for data to be available for analysis. | | | | | |
