# Supplementary material for: Electronic cigarette use during pregnancy and the risk of adverse birth outcomes: A cross-sectional surveillance study of the US Pregnancy Risk Assessment Monitoring System (PRAMS) population
Source: PLoS One. 2023 Oct 24;18(10):e0287348. doi: 10.1371/journal.pone.0287348 (PMC10597477; doi:10.1371/journal.pone.0287348)
Supplement: S2 Table — (DOCX) [file pone.0287348.s010.docx]

| **S2 Table. Measurement of cigarette use in the PRAMS Survey, 2016-2020.**   \| **Combustible Cigarette Use** \| \| \| --- \| --- \| \| Have you smoked any cigarettes in the past 2 years? \| No (skip follow-up questions)  ***Yes*** (continue to follow-up questions) \| \| In the 3 months before you got pregnant, how many cigarettes did you smoke on an average day? \| - I didn’t smoke then. - Less than 1 cigarette - 1 to 5 cigarettes - 6 to 10 cigarettes - 11 to 20 cigarettes - 21 to 40 cigarettes - 41 cigarettes of more \| \| In the last 3 months of your pregnancy, how many cigarettes did you smoke on an average day? \| \| How many cigarettes do you smoke on an average day now? \| \| **Electronic Cigarette & Nicotine Products Use** \| \| \| Have you used e-cigarettes or other electronic nicotine products in the past 2 years? \| No (skip follow-up questions)  ***Yes*** (continue to follow-up questions) \| \| During the 3 months before you got pregnant, on average, how often did you use e-cigarettes or other electronic nicotine products? \| - I did not use e-cigarettes or other electronic nicotine products then - 1 day a week or less - 2-6 days a week - Once a day - More than once a day \| \| During the last 3 months of your pregnancy, on average, how often did you use e-cigarettes or other electronic nicotine products? \|   PRAMS, Pregnancy Risk Assessment Monitoring System |
| --- | --- | --- | --- | --- | --- | --- | --- | --- | --- | --- | --- | --- | --- | --- | --- |
