# Supplementary material for: Electronic cigarette use during pregnancy and the risk of adverse birth outcomes: A cross-sectional surveillance study of the US Pregnancy Risk Assessment Monitoring System (PRAMS) population
Source: PLoS One. 2023 Oct 24;18(10):e0287348. doi: 10.1371/journal.pone.0287348 (PMC10597477; doi:10.1371/journal.pone.0287348)
Supplement: S3 Table — (DOCX) [file pone.0287348.s011.docx]

**S3 Table. Frequency and proportion of subjects missing in one or more covariates in the population of women who gave live singleton births in 2016-2020, PRAMS.**

|  | Non-use (N = 173,574)^a^ | | EC only use (N = 977)^a^ | | | CC only use (N = 14,752)^a^ | | | Dual use (N = 1,404)^a^ | | | | Total (N = 190,707)^a^ | | | |
| --- | --- | --- | --- | --- | --- | --- | --- | --- | --- | --- | --- | --- | --- | --- | --- | --- |
|  | No.^a^ | % (95%CI)^b^ | No.^a^ | % (95%CI)^b^ | | No.^a^ | | % (95%CI)^b^ | No.^a^ | | | % (95%CI)^b^ | No.^a^ | | | % (95%CI)^b^ |
| Maternal age at delivery | 6 | 0.00 (0.00, 0.00) | 1 | 0.25 (0.03, 1.72) | 1 | | 0.00 (0.00, 0.01) | | | 0 | 0.00 (0.00, 0.00) | | | 8 | 0.00 (0.00, 0.01) | |
| Maternal race/ethnicity | 1197 | 0.79 (0.73, 0.86) | 3 | 0.36 (0.08, 1.70) | 101 | | 0.61 (0.43, 0.85) | | | 9 | 0.64 (0.21, 1.95) | | | 1310 | 0.78 (0.72, 0.84) | |
| Maternal education | 1528 | 0.80 (0.74, 0.87) | 8 | 0.55 (0.18, 1.61) | 156 | | 0.80 (0.60, 1.06) | | | 11 | 0.51 (0.17, 1.56) | | | 1703 | 0.80 (0.74, 0.86) | |
| Marital status | 91 | 0.04 (0.03, 0.05) | 5 | 0.30 (0.08, 1.05) | 44 | | 0.17 (0.10, 0.31) | | | 7 | 0.10 (0.04, 0.25) | | | 147 | 0.05 (0.04, 0.06) | |
| Household income | 14928 | 8.74 (8.53, 8.95) | 74 | 7.60 (5.36, 10.67) | 1174 | | 7.47 (6.78, 8.22) | | | 76 | 5.59 (3.85, 8.05) | | | 16252 | 8.63 (8.42, 8.83) | |
| Maternal WIC program participation | 2367 | 1.32 (1.24, 1.40) | 16 | 0.80 (0.36, 1.77) | 235 | | 1.52 (1.21, 1.92) | | | 24 | 1.16 (0.56, 2.37) | | | 2642 | 1.33 (1.25, 1.41) | |
| Pregnancy intention | 0 | NA^c^ | 0 | NA | 0 | | NA | | | 0 | NA | | | 0 | NA | |
| Kotelchuck index | 4992 | 2.51 (2.40, 2.63) | 34 | 2.90 (1.75, 4.76) | 429 | | 2.50 (2.11, 2.96) | | | 34 | 1.25 (0.66, 2.38) | | | 5489 | 2.51 (2.40, 2.62) | |
| Prenatal care started in the 1^st^ trimester of pregnancy | 3702 | 2.02 (1.92, 2.12) | 24 | 2.20 (1.14, 4.23) | 365 | | 2.13 (1.78, 2.55) | | | 38 | 1.89 (1.18, 3.01) | | | 4129 | 2.03 (1.93, 2.13) | |
| Parity | 342 | 0.19 (0.16, 0.23) | 4 | 0.40 (0.11, 1.43) | 32 | | 0.25 (0.15, 0.44) | | | 5 | 0.27 (0.06, 1.31) | | | 383 | 0.20 (0.17, 0.23) | |
| History of preterm birth | 353 | 0.22 (0.18, 0.25) | 1 | 0.05 (0.01, 0.35) | 43 | | 0.21 (0.11, 0.38) | | | 3 | 0.22 (0.06, 0.73) | | | 400 | 0.21 (0.18, 0.25) | |
| Pre-pregnancy BMI | 3741 | 2.31 (2.20, 2.43) | 22 | 1.32 (0.64, 2.68) | 458 | | 3.12 (2.64, 3.69) | | | 47 | 3.71 (2.30, 5.93) | | | 4268 | 2.37 (2.26, 2.48) | |
| Pre-pregnancy multivitamin use per week | 929 | 0.62 (0.55, 0.69) | 7 | 1.71 (0.58, 4.91) | 56 | | 0.26 (0.17, 0.39) | | | 3 | 0.38 (0.07, 2.02) | | | 995 | 0.60 (0.54, 0.66) | |
| Pre-pregnancy alcoholic drinks per week | 1209 | 0.77 (0.70, 0.84) | 9 | 0.63 (0.24, 1.63) | 135 | | 0.71 (0.54, 0.93) | | | 15 | 1.22 (0.51, 2.90) | | | 1368 | 0.76 (0.70, 0.83) | |
| Delivery method | 136 | 0.10 (0.08, 0.13) | 1 | 0.02 (0.00, 0.10) | 14 | | 0.04 (0.02, 0.09) | | | 1 | 0.01 (0.00, 0.05) | | | 152 | 0.10 (0.08, 0.12) | |
| Year of delivery | 0 | NA | 0 | NA | 0 | | NA | | | 0 | NA | | | 0 | NA | |
| Residence | 0 | NA | 0 | NA | 0 | | NA | | | 0 | NA | | | 0 | NA | |

^a^Unweighted sample size.

^b^Weighted prevalence and corresponding confidence interval (expressed as a percentage).

^c^No missing.
