# Supplementary material for: Electronic cigarette use during pregnancy and the risk of adverse birth outcomes: A cross-sectional surveillance study of the US Pregnancy Risk Assessment Monitoring System (PRAMS) population
Source: PLoS One. 2023 Oct 24;18(10):e0287348. doi: 10.1371/journal.pone.0287348 (PMC10597477; doi:10.1371/journal.pone.0287348)
Supplement: S4 Table — (DOCX) [file pone.0287348.s012.docx]

**S4 Table. Frequency and proportion of subjects missing in one or more covariates in the population of women who all used ECs prior to pregnancy and gave live singleton births in 2016-2020, PRAMS.**

|  | Continued use (n=2,002^a^) | | Quit use (n=5,875^a^) | | Overall | |
| --- | --- | --- | --- | --- | --- | --- |
|  | No.^a^ | % (95%CI)^b^ | No.^a^ | % (95%CI)^b^ | No.^a^ | % (95%CI)^b^ |
| Maternal age at delivery | 1 | 0.13 (0.02, 0.91) | 1 | 0.00 (0.00, 0.01) | 2 | 0.03 (0.01, 0.22) |
| Maternal race/ethnicity | 9 | 0.57 (0.21, 1.52) | 39 | 0.53 (0.33, 0.86) | 48 | 0.54 (0.35, 0.84) |
| Maternal education | 18 | 0.60 (0.27, 1.36) | 44 | 0.56 (0.36, 0.87) | 62 | 0.57 (0.38, 0.84) |
| Marital status | 11 | 0.21 (0.08, 0.56) | 8 | 0.08 (0.03, 0.19) | 19 | 0.11 (0.06, 0.21) |
| Household income | 129 | 7.00 (5.33, 9.16) | 380 | 6.65 (5.69, 7.76) | 509 | 6.74 (5.89, 7.70) |
| Maternal WIC program participation | 35 | 0.95 (0.51, 1.76) | 71 | 0.84 (0.58, 1.23) | 106 | 0.87 (0.63, 1.20) |
| Pregnancy intention | 0 | NA^c^ | 0 | NA | 0 | NA |
| Kotelchuck index | 60 | 2.02 (1.33, 3.07) | 159 | 2.70 (2.12, 3.42) | 219 | 2.53 (2.05, 3.11) |
| Prenatal care started in the 1^st^ trimester of pregnancy | 57 | 1.95 (1.25, 3.02) | 88 | 1.94 (1.43, 2.61) | 145 | 1.94 (1.51, 2.49) |
| Parity | 8 | 0.25 (0.08, 0.74) | 14 | 0.31 (0.13, 0.70) | 22 | 0.29 (0.15, 0.58) |
| History of preterm birth | 3 | 0.14 (0.04, 0.48) | 12 | 0.14 (0.05, 0.38) | 15 | 0.14 (0.06, 0.31) |
| Pre-pregnancy BMI | 61 | 2.45 (1.65, 3.63) | 114 | 1.95 (1.43, 2.65) | 175 | 2.07 (1.62, 2.65) |
| Pre-pregnancy multivitamin use per week |  |  | 19 | 0.21 (0.12, 0.37) | 28 | 0.44 (0.24, 0.83) |
| Pre-pregnancy alcoholic drinks per week | 24 | 1.23 (0.65, 2.33) | 33 | 0.43 (0.26, 0.71) | 57 | 0.63 (0.42, 0.94) |
| Delivery method | 9 | 1.16 (0.46, 2.86) | 2 | 0.01 (0.00, 0.03) | 3 | 0.01 (0.00, 0.02) |
| Maternal CC use prior to pregnancy | 26 | 1.51 (0.81, 2.81) | 53 | 0.96 (0.62, 1.49) | 79 | 1.10 (0.76, 1.57) |
| Year of delivery | 0 | NA | 0 | NA | 0 | NA |
| Residence | 0 | NA | 0 | NA | 0 | NA |

^a^Unweighted sample size.

^b^Weighted prevalence and corresponding confidence interval (expressed as a percentage).

^c^No missing.
